# How COVID-19 affects voting for incumbents: Evidence from local elections in France

**DOI:** 10.1371/journal.pone.0297432

**Published:** 2024-03-19

**Authors:** Davide Morisi, Héloïse Cloléry, Guillaume Kon Kam King, Max Schaub

**Affiliations:** 1 Department of Political Science and Public Management, University of Southern Denmark, Odense, Denmark; 2 Collegio Carlo Alberto, Turin, Italy; 3 CREST, École Polytechnique, IP Paris, Palaiseau, France; 4 INRAE, Université Paris-Saclay, Jouy-en-Josas, France; 5 Department of Political Science, University of Hamburg, Hamburg, Germany; 6 WZB Berlin Social Science Center, Berlin, Germany; University of Glasgow, UNITED KINGDOM

## Abstract

How do voters react to an ongoing natural threat? Do voters sanction or reward incumbents even when incumbents cannot be held accountable because an unforeseeable natural disaster is unfolding? We address this question by investigating voters’ reactions to the early spread of COVID-19 in the 2020 French municipal elections. Using a novel, fine-grained measure of the circulation of the virus based on excess-mortality data, we find that support for incumbents increased in areas that were particularly hard hit by the virus. Incumbents from both left and right gained votes in areas more strongly affected by COVID-19. We provide suggestive evidence for two mechanisms that can explain our findings: an emotional channel related to feelings of fear and anxiety, and a prospective-voting channel, related to the ability of incumbents to act more swiftly against the diffusion of the virus than challengers.

## 1. Introduction

Natural disasters influence voting. After catastrophic events—such as earthquakes, floods, and hurricanes—voters often reward political candidates and incumbents [1–5] or punish them electorally [6–11], depending on how they responded to such events. Virtually all available evidence focuses on how voters *retrospectively* evaluate politicians’ performance after natural disasters have occurred [12], and whether these evaluations influence voting. However, we do not know how voters respond to a threatening event *while* it is unfolding. How do voters react to an ongoing natural threat? Do voters sanction or reward incumbents even when incumbents cannot be held accountable because an unforeseeable natural disaster is still unfolding?

We address this question by investigating voters’ reactions to the early spread of COVID-19 in France. COVID-19 differs from other natural disasters since it is a prolonged threat that does not result in physical destruction but spreads invisibly, potentially affecting all human beings. Like other infectious diseases [13], COVID-19 generates fear, which, we argue, increases voters’ tendency to support incumbent candidates as a means to obtain safety and security. This dynamic applied especially at the onset of the pandemic when there was uncertainty regarding the best strategy for containing the virus and guaranteeing citizens’ safety.

We focus on the municipal elections that took place in France on 15 March 2020, when SARS- Cov-2, the virus causing COVID-19, was already circulating in the country. To estimate the “threat” of COVID-19, we developed a novel, fine-grained measure of the spread of the virus (the “prevalence” of COVID-19) at the municipality level, using excess-mortality data weighed by the age and sex structure of the population. This measure accounts for the Infection Fatality Ratio [14] and allows us to address the problem of underreporting with regard to the circulation of the virus at the beginning of the pandemic [15]. Even if the total number of *confirmed* cases of COVID-19 in France on 15 March 2020 was relatively low—a total of 4532 [16]—our estimates indicate that a substantial share of French voters already knew of other people who suffered from COVID-19 in their local area. Specifically, we estimate that there was a small but sizeable group of municipalities (7 percent of the total), in which a voter had at least a 30 percent probability of knowing at least another person with COVID-19 symptoms in their inner circle of friends and acquaintances. Even if the proportion of people with COVID-19 symptoms in a given municipality is low, the probability that a random voter knows at least one person with COVID-19 symptoms can be large (as further explained below).

We exploit the uneven diffusion of the virus in the country to test whether support for local incumbents increased in municipalities that were particularly hard hit by COVID-19. In particular, we estimate the effect of being severely affected using a) regression models that control for the lagged dependent variable and condition on baseline mortality rates, b) a difference-in-differences approach, and c) propensity score matching. In line with evidence for a “rally-around-the-flag” effect at the beginning of the pandemic [17–22], our findings show that vote shares for local incumbents increased as the threat of COVID-19 increased. In municipalities with a high circulation of the virus (tenth decile), the average vote share for incumbents was 2.5 percentage points higher than in places with a low circulation (first decile). The effects are insensitive to the political affiliation of the incumbents, meaning that support for incumbents increases regardless of whether they are left-wing, right-wing, or from other parties.

We also show that the effect of COVID-19 on voting is more pronounced in areas a) where people feel particularly anxious about the pandemic, and b) where economic welfare improved during the outgoing administration. This evidence supports our argument that, when faced with the circulation of the virus, voters, on the one hand, turned to incumbents as a means of reducing fear and anxiety–an “emotional channel” in line with evidence from the United States [23, 24]. In addition, following the logic of prospective voting, we argue that voters support incumbents to maintain continuity with the current administration as the most efficient means of tackling the virus, especially in areas where the incumbent mayors had performed well.

In the next section, we review existing research on the effects of natural disasters on voting, and advance our arguments for why the early spread of COVID-19 should increase support for local incumbents. We then provide some brief background information on the 2020 municipal elections in France, describe our data and methods, and present the results. In the conclusion, we summarize our findings and discuss the implications and the limitations of our study.

## 2 Natural threats and support for incumbents

Research shows that voters punish or credit political candidates depending on how these candidates respond to severe weather phenomena. For example, studies indicate that incumbent politicians lose support if their response to natural disasters is perceived as inadequate [6, 8, 11, 25]. Voters also punish incumbents in the aftermath of severe weather events that cause economic damage, such as tornadoes, floods and extreme rainfall [4, 7, 9, 10], and even in the case of events that are clearly out of politicians’ control, such as shark attacks [26]. On the other hand, there is evidence that voters reward governments and incumbent politicians who are seen as having responded well to natural disasters [1–3, 5, 12].

The primary mechanism describing voters’ reaction *after* a disaster has occurred is retrospective voting [27]: on election day, voters sanction or reward politicians depending on how well they responded to the damage caused by the natural catastrophe. At the beginning of the COVID-19 pandemic, however, it was unclear how to best respond to the circulation of the virus. As an invisible threat that does not lead to physical destruction and persists for an indefinite period, COVID-19 cannot be tackled with the same means used after a natural catastrophe. Relief-spending and the types of reconstruction undertaken after natural disasters were not viable options for incumbent politicians at the beginning of the pandemic. This is especially true for local mayors who lacked the legal authority to introduce strong containment measures, such as lockdowns. Thus, the initial spread of COVID-19 should not have affected voting for local incumbents through retrospective evaluations since it was unclear how politicians ought to have responded to the pandemic.

In the absence of retrospective evaluations, threatening events can still induce people to express greater support for incumbent political leaders via emotional reactions, the so-called ‘rally-around- the-flag’ effect [28–30]. This effect has been identified in relation to terrorist attacks (see for example [31–35]) and military crises [28], both of which have been found to lead to a boost in presidential popularity and government support. Recent studies have argued that the threat of COVID-19 should have similar rally effects [21], at least at the beginning of the pandemic. Indeed, evidence indicates that support for world leaders [17–19, 22] and trust in governments [20, 36–38] increased at the beginning of the COVID-19 pandemic. We do not know, however, whether approval ratings actually translated into voting behaviour (but see [39]), and it is unclear what mechanisms could connect the spread of the virus to voting behaviour.

We argue that the early spread of COVID-19 should lead voters to increase support for local incumbents mainly through two channels. Similar to rally effects, the first channel is emotional, although involving a different type of emotion: anxiety (instead of anger or patriotic arousal). It has been argued that rally effects are activated mostly by a sentiment of patriotism and by anger towards a clearly identifiable external aggressor, such as a terrorist group [29, 40]. The threat of COVID-19, however, should not activate the same feelings of anger and patriotism, since there is no deliberate attack behind the circulation of the virus (i.e., the virus did not “intend” to attack a population, as terrorists do), and there is no clearly identifiable human enemy against whom the population should mobilize.

Instead, we argue that COVID-19 triggers fear and anxiety, as indicated by psychological studies [41, 42] and by recent evidence in the U.S. [23] (see also [21]). As with the Ebola virus when it reached the US in 2014 [13], at the beginning of the pandemic, COVID-19 represented an invisible threat against which there was no clear remedy, thus potentially inducing fear in the population. Different strands of theories indicate that sentiments of fear and anxiety lead to risk-aversion (for a review, see [43]), which, in turn, should translate into increased support for incumbent candidates, as they represent a safer, status quo option compared to the risk of electing an as-yet-untested challenger.

Furthermore, according to terror-management [44] and motivated-social-cognition theory [45, 46], people want to see the world as a secure place. Therefore, in the presence of a fear-inducing threat such as COVID-19, voters should turn their support towards incumbent politicians who offer an actual or symbolic sense of safety and security because they represent the status quo. Indeed, evidence from the United States indicates that the spread of COVID-19 and lockdown measures at the beginning of the pandemic increased support for candidates representing the “status quo” [24].

Although these different theoretical backgrounds speak in favour of a positive association between the circulation of COVID-19 and support for incumbent candidates, a negative association can also be conceivable for two different reasons. First, given accumulated evidence that “fear, threat, and anxiety often contribute to right-wing *extremism*” [47], it is possible that the spread of COVID-19 shifts voters away from incumbents, if incumbents are predominantly not extremists. Although this theoretical possibility is plausible, it seems highly unlikely to occur in the context of local elections in France, where the vote share for extreme right-wing candidates is negligible.

Second, it is conceivable that where the virus circulates more, it is mostly risk takers who go to vote, because risk-averse voters decide to stay home in the first place. Thus, in these areas we should observe a decline in both turnout and vote for incumbents. Although this theoretical channel is also plausible, it rests on the assumption that risk takers are particularly likely to choose challengers over incumbents. However, it is also possible that risk takers prefer incumbents over challengers for purely instrumental reasons related to the prospective channel described below, in line with evidence that instrumental risk taking is linked to more rational decisions [48] and that risk takers “tend to decide more strictly on the basis of cost-benefit considerations” [49].

The discussion of these alternative scenarios leads to the second channel related to a simple version of prospective voting, that is, voting on the basis of how candidates will perform in the upcoming administration. This channel is based on the simple consideration that during a crisis “it is not a good time for a change.” In line with the idea of prospective voting [39, 50, 51], voters compare how challengers versus incumbents will perform in relation to containing the virus. Anticipating that the threat of COVID-19 is going to last, and given that virtually no candidate has a track record in dealing with a pandemic, voters might consider that incumbents will perform better simply because they have had several years of experience with the local administration and can swiftly mobilize all the available local resources. For instance, French mayors had to cooperate with existing local actors (schools, local NGOs, and volunteers) during the pandemic to share information, distribute masks, or coordinate with other administrations. Voters could interpret the politicians’ ability to work with local actors as a signal of better future management of the crisis. Because they already knew these local actors, the incumbents probably had an advantage over the other candidates. For these reasons, in a scenario of a looming threat, voters might prefer to maintain continuity with the current administration as the most efficient means of tackling the virus and restoring safety. On the other hand, choosing a challenger over the incumbent might delay the necessary response to the pandemic due to the period of transition to the new administration. Furthermore, voters might simply consider that in a situation of high uncertainty produced by the circulation of the virus, electing a challenger would add further uncertainty regarding how the local administration will deal with the pandemic. Thus, they might prefer incumbents as a safer way of preserving public safety.

Both the emotional channel and the prospective-voting channel lead us to the expectation that support for incumbents should increase in areas where the spread of COVID-19 was higher at the early stage of the pandemic. According to these theoretical channels, support for incumbents should increase regardless of their political affiliation, as voters threatened by COVID-19 turn to incumbents mainly because they represent the status quo. This said, previous studies indicate that it is mostly right-wing incumbents that gain from military threats because their aggressive defense strategies are seen as more in line with citizens’ need for protection compared to other parties’ strategies [52, 53]. Although the spread of COVID-19 differs from military threats, if right-wing incumbents are better suited to reducing anxiety and addressing citizens’ need for safety and security, we might expect the effect to be strongest for right-wing incumbents. This expectation is also in line with cited evidence that fear and anxiety contribute to a shift towards right-wing political attitudes (for a review, see [47]).

## 3 Municipal elections in France

On March 15, 2020 French voters went to the polls to choose the mayors of all of the nearly 35,000 municipalities in the country. Municipal elections in France are held every six years, and, depending on the size of the municipality, consist of one or up to two rounds. In small municipalities with under 1,000 inhabitants, voters choose lists of candidates and are allowed to cast a vote for more than a single candidate in a majoritarian system. In addition, the candidates in towns with less than 1000 inhabitants are not required to declare any political affiliation. In larger towns and cities with more than 1,000 inhabitants, elections consist of one or two rounds. In the first round, all lists of candidates standing for the election compete against each other. In case no list obtains more than 50 percent of the vote, a run-off election is held in which all the lists that obtained more than 10 percent of the votes compete again for election. This second round is usually held two weeks after the first round.

In 2020 the first round of the municipal elections was held as scheduled, despite a partial lockdown in some places. However, as the number of cases of COVID-19 continued to increase, the day after the first round of the elections, President Macron announced that the second round would be postponed to an unspecified date, which was later set to June 28, 2020. Compared to the previous local elections in 2014, turnout dropped substantially. While in 2014, 63.5 percent of eligible voters went to cast their ballot in the first round of the elections, on March 15, 2020 less than half of eligible voters (44.7 percent) went to vote. This drop in participation was likely due to the circulation of the virus, as confirmed by recent studies [54–56].

## 4 Data and methods

### 4.1 Voting and census data

We retrieve electoral data for the municipal elections in 2020 and 2014 from the Ministry of the Interior [57–59]. We consider only the municipalities that in both election rounds had more than 1000 inhabitants due to the differences in electoral rules explained above. We also exclude overseas territories and the cities of Paris, Marseille and Lyon, since the voting system also differs in these areas. After excluding these observations, we are left with a sample of 8,193 municipalities.

To determine which candidates running in 2020 were incumbents, we matched the names of those who were elected mayor in 2014 with the names of the 2020 candidates. This operation gives us our effective sample of 4,952 municipalities (around 56%) in which an incumbent was running in 2020. Balance statistics indicate that municipalities with and without incumbents are statistically indistinguishable across all our indicators, apart from population density and turnout in 2014, as municipalities with an incumbent are more densely populated and have a higher turnout in 2014 than municipalities without an incumbent (see S1 Table in S1 File). Our outcome of interest is the share of votes for the incumbents in the first round of the municipal elections in 2020. Crucially, in all regression models we include the vote share for the same candidates in 2014, which allows us to estimate whether the spread of COVID-19 influences the *change* in vote shares for incumbents in 2020 compared to 2014.

We also retrieve information about the political affiliation of the incumbent candidates to test for heterogeneous effects depending on the incumbents’ political leaning. In 2014, all candidates competing in municipalities above 1000 inhabitants had to indicate their political affiliation, which was then classified by the Ministry of the Interior. We use this information to distinguish between incumbents related to left-wing parties (22%), right-wing parties (47%), and a residual category including mostly candidates with no clear left-right affiliation and a few centrist candidates (31%). In S3 Table (S1 File) we provide detailed information about each political group.

Furthermore, we collect census data at the municipality level from the French national institute for statistics for the year 2017 to control for key socio-economic indicators that might confound the relationship between the spread of COVID-19 and voting for incumbents [60]. Specifically, in each model we include measures of population density, male over female ratio, the share of people aged above 65, the share of immigrants, the share of blue-collar workers, the share of unemployed inhabitants, the median income of households in the municipality, and the share of people with a junior high school degree (“CAP” or “BEP” degree) and with a bachelor’s degree. We use the logged transformation for each of these variables (apart from the median income) in order to account for the presence of clear outliers. Furthermore, we control for the level of turnout in 2014 to account for imbalances in voters’ electoral participation at the municipality level, and for the number of candidates running in the first round of the elections. The latter variable is particularly important, since the incumbent vote share is partially determined by the level of electoral competition in a given municipality (with higher vote share where fewer candidates compete). A directed acyclic graph (DAG) justifying the choice of control variables is included in the supplementary S2 Fig in S1 File. For summary statistics, see S2 Table in S1 File.

### 4.2 Estimating the spread of COVID-19

Our core independent variable is a municipality’s degree of affectedness by COVID-19. As no such measure was readily available, we estimated it ourselves using standard epidemiological methods. The aim of this section is to provide an overview of how this measure is constructed (additional technical details are available in Appendix C (S1 File)). Specifically, we sought to construct a fine-grained measure of the spread of Covid-19 at the municipality level at the time of the election, which we denote *Cov*_*m*,20_. This quantity is then used as an independent variable to predict the vote share received by incumbents, with the aim to measure the impact of COVID-19 on voting behaviour.

Estimating the spread of COVID-19 at the municipality level is challenging because of scattered data availability and the low reliability of common measures. For instance, reported cases of COVID- 19 at the beginning of the pandemic were sensitive to differences in testing frequencies across the country and underestimated the circulation of the virus [15], while data about hospitalisations are not available at the municipality level since hospitals are located only in a few cities. To estimate the spread of COVID-19 at the municipality level, we therefore utilized exhaustive mortality records at the municipality level to estimate COVID-19 prevalence in each municipality at the time of the election. We took into account the differential probability of dying depending on age and sex (the Infection Fatality Ratio (IFR, [14])), which essentially weights the excess mortality data by the age and sex structure of the population at the municipality level.

More precisely, we first computed the excess mortality (or excess hazard) at the municipality level by collecting all mortality records between 2015 and 2020 [61]. We estimated excess mortality around the time of the first round of elections by comparing mortality figures to those of the same period 2015–2019, thus avoiding issues of seasonal variability. Excess mortality has been presented as a reliable method to estimate the number of COVID-19 cases because mortality appears fairly stable over a period of a few years, making extreme mortality events clearly visible in the form of peaks [62, 63]. Yearly influenza outbreaks, for instance, are clearly identifiable in country-wide weekly mortality records (see S4 Fig in S1 File), while aggregated mortality rates seem reasonably stable over the last half-decade for the period considered (see S5 Fig in S1 File). Given that there is a delay between infection and death, we consider all deaths occurring between election day (March 15, 2020) to six weeks later, which should provide information about the number of infected at the time of the election. There is a risk of simultaneity bias: (i) the spread of the disease can affect the vote share for incumbents, but (ii) turnout—and thus vote shares for incumbents—itself can affect the spread of COVID-19 [64]. In a supplementary analysis, we consider a shorter time window of four weeks, less likely to be affected by the simultaneity issue. We obtain substantially similar results with this four-week window (see S7 Table in S1 File).

Scholars have argued that excess death may be related indirectly to COVID-19 through the saturation of hospital facilities, interruption of preventative programmes or the impact of social restrictions on physical and mental health [62]. However, it seems unlikely that these factors played a strong role at the very beginning of the pandemic. Instead, researchers have argued that the probability of dying from COVID-19 once infected by the virus (Infection Fatality Ratio, IFR) is heavily determined by age and sex [14] and that excess mortality should be weighed by these factors if we intend to use it as a proxy for the circulation of the virus. In this sense, a 5% excess mortality in a relatively young city indicates that SARS-Cov-2, the virus causing COVID-19, is circulating *more* than in a relatively old city with the same level of excess death since young people are less likely to die from COVID-19 than elderly people. Therefore, we build a model to estimate COVID-19 prevalence (the proportion of the population infected by COVID-19) at the municipality level from mortality data and use it as a novel measure describing the spread of COVID-19, which weights excess death by age and sex-specific infection fatality ratios to estimate the number of cases more precisely.

Specifically, we use a type of Poisson mortality model where the municipality-level baseline hazard over the 6 weeks period is considered constant between 2015 and 2019 and death counts in 2020 result from the baseline hazard plus an excess hazard due to COVID-19. Excess hazard is related to prevalence modulated by the age and sex-specific Infection Fatality Ratio from [14]. We obtain a municipality-level measure of prevalence, along with age and sex-specific baseline hazards for each municipality. Population data is obtained from Census data from 2010 to 2017 [65] and then either linearly or log-linearly extrapolated for 2018–2020 (see details in Appendix C.2 (S1 File)). To deal with the presence of outliers among the municipalities, in the regression models we use either a decile version (treated as a continuous variable) or a quartile version of our measure (to test for non-linear effects). In supplementary analyses, we show that substantially similar results are obtained using a logged transformation or a quintile version of our measure (see S4 and S5 Tables in S1 File).

Although in the majority of French municipalities the SARS-Cov-2 virus was either not present or barely spreading on election day on 15 March 2020, we estimate that in a small but sizeable number of cities voters had a relatively high probability of knowing other people with COVID-19 symptoms. Assuming that the average voter knows around 15 people, and assuming homogeneity among voters, based on our prevalence measure we estimate that on 15 March 2020 there were 654 cities (7 percent of the total) and 255 cities (3 percent of the total) in which a person had a probability of at least 30 percent or 50 percent respectively of knowing at least one person with COVID-19 symptoms in their circle of friends and acquaintances. This is a very conservative estimate, considering a small social network of 15 people instead of the network of 150 people calculated by Dunbar [66], to account for the fact that not all COVID-19 infected people may experience symptoms or share that information in their social network. This stems from the fact that even if only few people suffer from COVID-19 in a municipality, the probability that a random voter knows at least one person with COVID-19 symptoms can be large—a counter-intuitive finding in line with the so-called “birthday paradox” [67, 68]. These basic estimates suggest that on election day there was already a substantial number of voters who were aware that the virus causing COVID-19 was circulating in their local area of residence.

### 4.3 Estimating the effect on incumbent voting

In order to estimate the effect of COVID-19 on incumbent vote shares, we estimate OLS regression models in the form

$$I_{m,d,20}=\alpha +\beta Cov_{m,20}+\gamma X_{c,t-1}+\eta I_{m,d,14}+\lambda_{d}+\epsilon_{m,d,t}$$ (1)

where *I*_*m*,*d*,20_ is the vote share received by the incumbent in a given municipality *m* in 2020; *Cov*_*m*,20_ measures the spread of COVID-19 in a given municipality in 2020; *X*_*c*,*t*−1_ is a vector including the municipality-level census variables presented above (measured in 2017), in addition to the level of turnout in 2014 and a measure of baseline mortality. The baseline mortality is the municipality-specific probability of dying during the period prior to the onset of the pandemic. The construction of both *Cov*_*m*,20_ and the baseline mortality was discussed in the previous section. Controlling for the baseline mortality is especially important since it captures various contributions to mortality—both observed and unobserved—thereby adjusting the otherwise quite diverse set of municipalities in terms of expected future mortality. *I*_*m*,*d*,14_ is the incumbent’s vote share in the election 2014, and *λ*_*d*_ are county (*Département*) fixed effects that remove time-invariant factors at this level from our estimations.

Our parameter of interest is β. Since our models include the lagged dependent variable (i.e., vote for incumbents in 2014), *β* measures the *change* in incumbent support between the two elections related to the spread of COVID-19, all else being equal. We opted for this specification because it allows for more flexibility in the way the independent variable can be specified. Below we show that our results also hold in a difference-in-differences framework, when matching municipalities using propensity scores and a punishing caliper, and are robust to a placebo test.

## 5 Results

**Fig 1:**
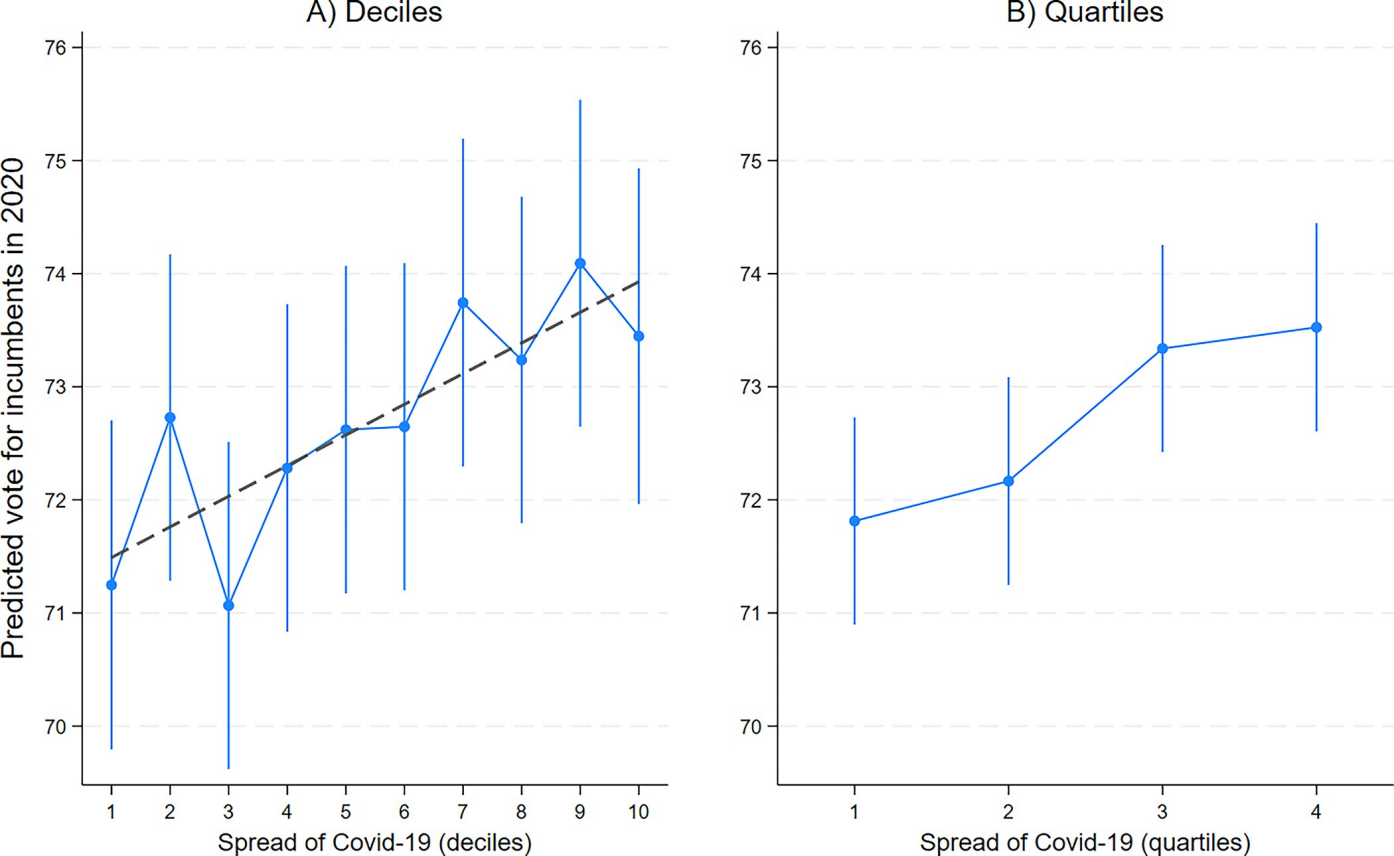
Effect of COVID-19 on vote for incumbents in 2020. *Note*: Coefficient plots from OLS regressions of vote for incumbents in 2020 on local spread of COVID-19 controlling for vote for incumbents in 2014 and additional municipality-level covariates. Vertical lines are 95% confidence intervals. Plot A: deciles of COVID-19 circulation treated either as categories (vertical lines) or as a linear predictor (dash line). Plot B: quartiles of COVID-19 circulation treated as categories. For complete regression estimates, see S4 Table in S1 File.

Fig 1 presents the results of our regression models. Our model explains a large part of the variation in votes for incumbents in 2020 (R-squared = 0.59). Support for incumbents in 2020 is higher in municipalities with higher population density, higher share of people with a bachelor’s degree, lower share of people with a junior high school degree, lower share of people aged above 65, and lower median income of households. As expected, vote share for incumbents increases as electoral competition decreases, as captured by the number of candidates running in each municipality (for complete results, see S4 Table in S1 File). Despite the high explanatory power of the regression model, our measure of the spread of COVID-19 has a positive effect: vote shares for incumbents are substantially higher where COVID-19 is more widespread.

The vertical bars in the left-hand plot of Fig 1 show the predicted vote share for incumbents in 2020 at each decile of COVID-19 prevalence, treated as a categorical predictor, holding all other factors constant. Despite variation, we observe a roughly linear increase moving from places with low to high circulation of COVID-19. If we treat COVID-19 prevalence as a linear predictor (the dotted line in the left-hand plot), we find that support for local incumbents is 2.5 percentage points higher in the municipalities with high circulation of the virus (tenth decile) than in the municipalities with low circulation (first decile)–an increase that is statistically significant at p*<*0.01. While in the latter group of municipalities the estimated, average vote share for local incumbents in the first round of the elections was 71.5 percent, in the former group it was 74 percent. If we use alternative model specifications, the difference between the tenth and the first decile of COVID-19 spread ranges between 1.9 and 4.8 percentage points (see S5 Table in S1 File).

If we divide our measure of the spread of COVID-19 in quartiles (right-hand plot of Fig 1), we find a substantial “jump” between the second and the third quartile, indicating that the effect of the virus on voting occurs mostly in the third and fourth quartiles compared to the first and second.

The average vote share for incumbents in the municipalities located in the third and fourth quartile of the distribution of COVID-19 spread is, respectively, 1.5 and 1.7 percentage points higher than the vote share in the municipalities with relatively low circulation (first quartile)–a difference that is statistically significant at p*<*0.05.

**Fig 2:**
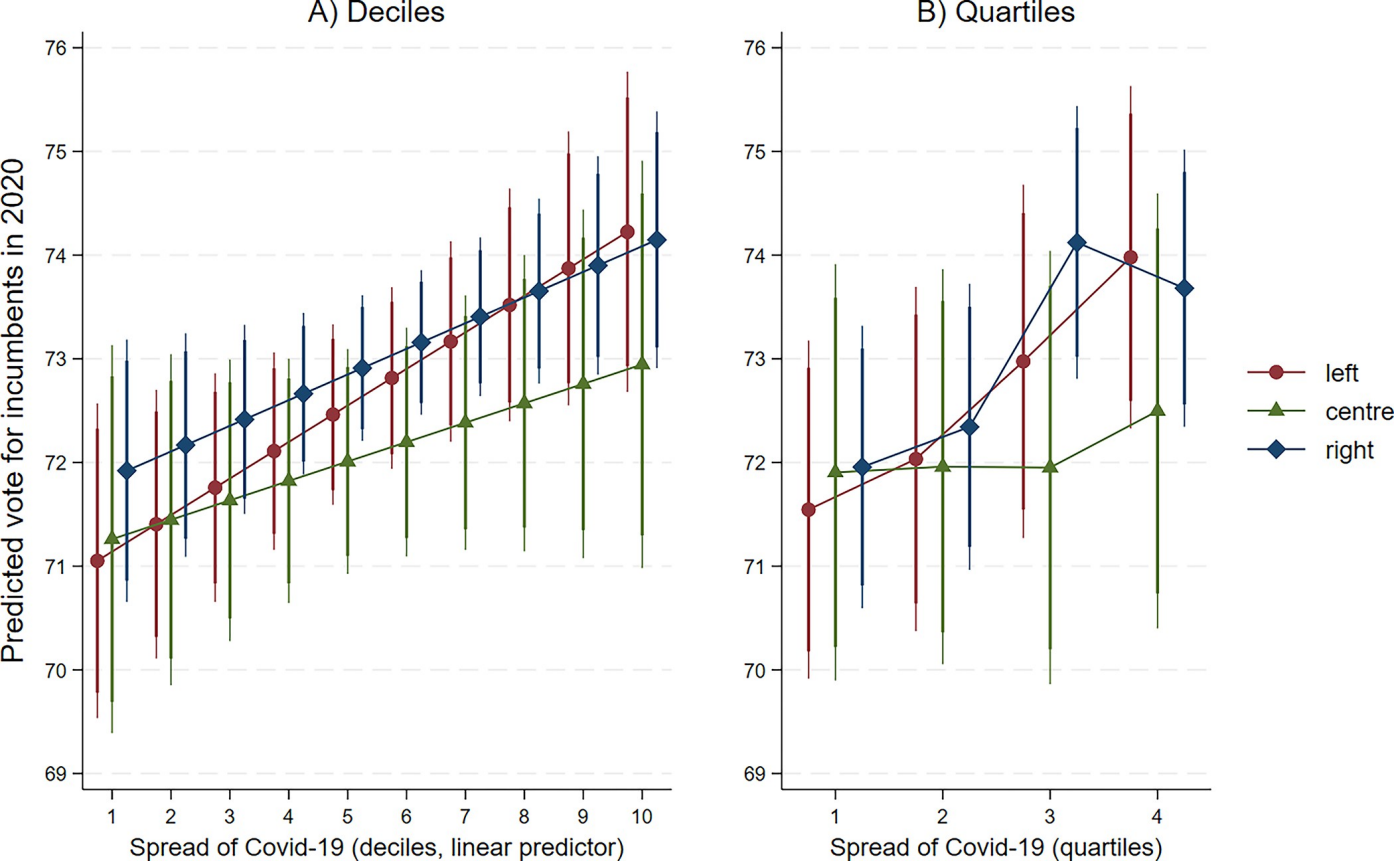
Effect of COVID-19 on vote for incumbents by political affiliation of incumbents. *Note*: Coefficient plots from OLS regressions of vote for incumbents in 2020 on local spread of COVID-19 interacted with the political affiliation of the incumbent (left-wing, right-wing or centrist). Vertical lines are 95% confidence intervals. Deciles of COVID-19 circulation treated as a linear predictor, and quartiles of COVID-19 circulation treated as categories. For complete regression estimates, see S8 Table in S1 File.

The effect of COVID-19 does not seem to vary substantially depending on the political affiliation of incumbents. If we interact our measure of the spread of the virus with a categorical variable indicating whether the incumbent is affiliated to a left-wing party, a right-wing party or another party, we do not find statistically significant interactions (see S8 Table in S1 File). Fig 2 plots the results of the interactions. The left-hand plot indicates that, if at all, the virus has a stronger effect on support for left-wing candidates (contrary to expectation), although there are no statistically significant differences between left-wing and right-wing candidates. The right-hand plot indicates that the “jump” in the COVID-19 effect between the second and the third quartile applies essentially to right-wing and left-wing candidates, while support for centrist candidates is less affected by the circulation of the virus. In sum, we cannot confirm previous studies showing that it is mostly right-wing candidates who gain support when voters are under threat [52, 53]. Instead, our results suggest that support for incumbents increased “across the board” in areas that were particularly hit by the virus.

### 5.1 Robustness checks and placebo tests

We implement alternative specifications of our models and conduct additional analyses to test the robustness of our results. First, we run the models using alternative specifications of our dependent variable and our measure of the spread of COVID-19. We find substantially similar results when a) we conduct first-difference regression models, in which the dependent variable is the difference in the incumbent vote share between 2020 and 2014 in each municipality (see S6 Table in S1 File); b) we use either a logged or a quintile version of our independent variable (see Models 3 and 4 in S4 Table (S1 File)), and c) we estimate the spread of COVID-19 using excess-mortality data registered four (instead of six) weeks after the first round of elections (see S7 Table in S1 File).

Second, we use propensity score matching to increase our confidence that municipalities with low and high prevalence of COVID-19 are statistically indistinguishable across all available indicators, apart from our measure of COVID-19. We divide our sample in treated and control observations, defining municipalities as ‘treated’ if they fall into the top two quartiles of municipalities affected by the spread of COVID-19. We then calculate the propensity score (the likelihood of a municipality being heavily affected by COVID-19) using the full set of control variables. Next, we match treatment and control observations with very similar propensity scores one-to-one applying a punishing caliper. Only matches whose propensity scores differ by no more than 0.025 points are retained. We then run our models on the sample of matched municipalities, and find essentially the same results: moving from the first to the tenth decile of COVID-19 spread is associated with an increase of 2.5 percentage points in votes for incumbents (see S9 Table in S1 File).

Third, we check if our results hold in a difference-in-differences (DiD) framework that compares within-municipality changes in incumbent vote shares across treatment and control municipalities over time. A ‘naive’ comparison of differences shows that the change in incumbent vote shares in treated vs. control municipalities between 2014 and 2020 differed by 1.8 percentage points (see S11 Table in S1 File). The estimate remains the same if we calculate the model in a regression framework using municipality fixed effects and controlling for changes in our control variables over time (see S12 Table in S1 File).

Fourth, we conduct placebo tests using vote shares for incumbents in 2014 as our outcome. We retrieved the list of mayors elected in the 2008 election from [69] to determine which candidates in 2014 were incumbents. Because of a change in the electoral system, there are only 533 municipalities in which the electoral system did not change between 2008 and 2014, and an incumbent was running in 2014. We then run the same models, using votes for incumbents in 2014 as dependent variable and including their vote shares in 2008 among the predictors. Reassuringly, we find no differences in support for incumbents in 2014 depending on the circulation of the virus in 2020 (see S10 Table in S1 File).

Lastly, we tested the parallel trend assumption, according to which the vote share for incumbents should change to a similar extent between the 2008 and the 2014 elections in municipalities with low and high spread of COVID-19. Given the change in the electoral system and the fact that not all mayors decided to run again in subsequent elections, there are only 204 municipalities in which a candidate was mayor in 2008 and 2014, and ran as an incumbent in 2020. A visual trend indicates a similar upward trend in the vote share for incumbents between 2008 and 2014 in municipalities with low and high spread of COVID-19 (S1 Fig, plot A in S1 File). Although the increase in municipalities with high spread of COVID-19 between 2008 and 2014 appears slightly steeper than the increase in municipalities with low spread of COVID-19 in the same period, regression models indicate that there is no statistically significant difference. We find, however, that votes for incumbents increased significantly in 2020 vs. 2014 in the municipalities with high spread of COVID-19, but not in the municipalities with low spread of COVID-19 (S1 Fig, plot C in S1 File). Importantly, we treat these results as merely indicative, given the very small sample of municipalities that we could use to perform the analysis.

### 5.2 Mechanisms

According to our theoretical framework, the effect of the early spread of COVID-19 on support for local incumbents can be explained by two channels: an emotional channel related to feelings of fear and anxiety, and a prospective-voting channel, related to the idea that the local administration led by the incumbent should be able to act more swiftly against the diffusion of the virus than a new administration led by a challenger. Although we cannot test these channels “directly” using aggregate data at the municipality level, we attempt to test them indirectly using additional data that can plausibly work as proxies for our mechanisms.

To test the emotional channel, we retrieved data from a national survey conducted by IPSOS, with a representative sample of 5,000 French adults between 7 and 14 April 2020 (data downloaded on 14 June 2020 from [70]). In the survey, the respondents were asked how frequently they “experienced a feeling of nervousness, anxiety or tension” or they were “unable to stop worrying or controlling your worries” in the past two weeks. As response categories, they could choose between “almost every day”, “more than half of the time”, “several days”, or “never”. We combine the responses to these two items into an additive index of perceived anxiety, rescaled from 0 (corresponding to “never experiencing anxiety”) to 1 (“experiencing it almost every day”). To the best of our knowledge, this is the only publicly available survey that measures feelings of anxiety on a large sample of voters at the beginning of the pandemic in France.

Although the respondents’ municipality of residence is not available in the survey, there is information about the county of residence and the size of the municipality of residence divided in five categories (“rural”, “between 2000 and 19,999 inhabitants”, “between 20,000 and 99,999”, “100,000 inhabitants or more”, “greater Paris”). Using this information, we grouped the respondents by county and municipality size, and then calculated the average level of perceived anxiety for all the respondents living in the municipalities of the same size within each county. With this operation we obtain 305 observations (geographical units) that we combine with our original dataset, matching by county and municipality size.

**Fig 3:**
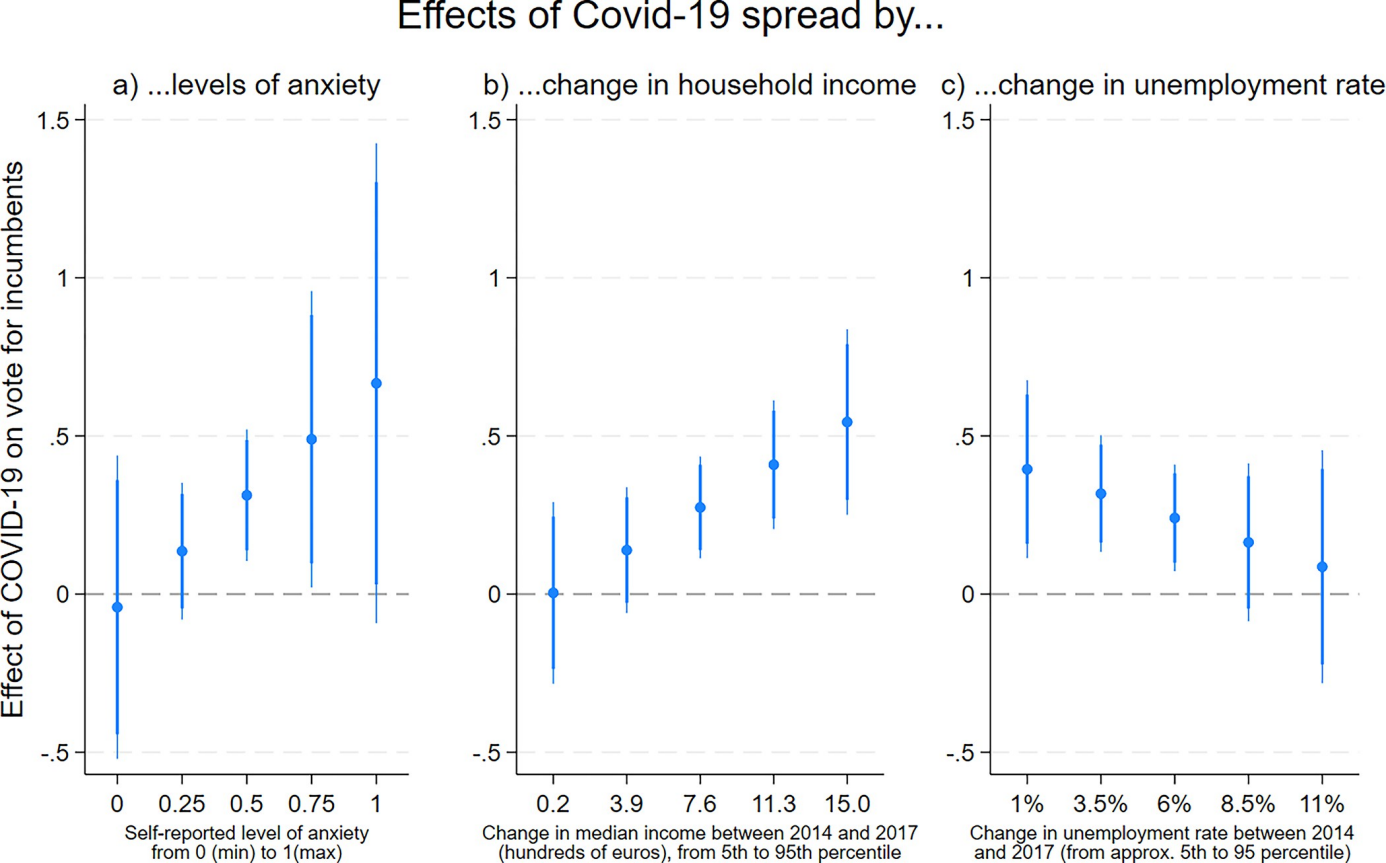
Testing mechanisms underlying the effect of COVID-19 (suggestive evidence). *Note*: The coefficients indicate the effect of one decile increase in the spread of COVID-19 on voting for incumbents in 2020 by interacted variables specified on the X-axes treated as continuous variables. Coefficients plots from OLS regressions controlling for vote for incumbents in 2014 and additional municipality-level covariates. Deciles of COVID-19 treated as linear predictor. Thin/thick lines are 95%/90% confidence intervals. Plot a: Effects by average levels of anxiety in 305 geographical units (municipalities of similar size within counties). For complete regression estimates, see S14 Table in S1 File.

We then introduce the aggregate measure of anxiety in our regression models and interact it with our measure of the spread of COVID-19, clustering standard errors at the level of municipality-size within county. If voters support incumbents as a means of reducing the fear and anxiety induced by the pandemic, we should find that votes for incumbents in 2020 increase especially where the circulation of the virus is high *and* people are particularly anxious about the pandemic. Our results suggests that this is the case. Although we do not find a statistically significant interaction, we find that there is no association between the circulation of the virus and voting for incumbents in areas where people (on average) do not feel anxious, while in areas with higher levels of anxiety this correlation is substantial (left-hand panel of Fig 3). This finding supports the idea that, under the threat of the virus, voters turn to incumbent candidates in search of safety and as a way to reduce fear and anxiety. In the absence of anxiety, the threat of COVID-19 does not affect voting.

Testing our second channel proves even more challenging, since we assume that voters (on average) would prefer incumbents over challengers to avoid the potential delays that might result from changing the political representatives of the local administration. Still, if this reasoning is correct, we can hypothesize that voters should be particularly prone to confirm incumbent mayors that have performed well during the last administration. The idea is that politicians who performed well in the former administration–even in domains that are unrelated to public healthcare–might also perform well in the next administration when facing the urgent task of dealing with the pandemic.

As indirect measures of past performance, we retrieve data about the median income and the unemployment rate at the municipality level in 2014, and compute the change between 2014 and 2017. If the median income increased and the unemployment rate decreased over time, voters might be particularly satisfied with their local mayors, even if these trends might be unrelated to the local administration’s performance. Thus, they should be particularly likely to vote for incumbents where the diffusion of COVID-19 is high and mayors have performed well in the past, since past performance can be used as an indicator of future performance against the pandemic.

To test this expectation, we introduced our measures of change in the regression models. We find a positive and statistically significant interaction between the change in median income and the spread of COVID-19 (see S14 Table in S1 File). As the centre-plot of Fig 3 shows, the association between the spread of COVID-19 and the vote for incumbents becomes substantial and statistically significant as median income increased between 2014 and 2017. The right-hand plot of Fig 3 shows the interaction between the change in the unemployment rate and the spread of COVID-19. Although the interaction term is not statistically significant, we find that the spread of COVID-19 influenced voting only in places where the unemployment rate decreased between 2014 and 2017.

The results presented so far are substantially the same if we treat our measure of COVID-19 spread as categories (quartiles) and display the effect of being in the fourth versus the first quartile of COVID-19 circulation (see S1 Fig in S1 File). Although these findings bring support to our theoretical mechanisms, they need to be treated with caution, since our measure of anxiety suffers from geographical limitations and we do not have a direct measure of the prospective-voting channel. In addition, we do not know whether the observed trends in local welfare change continued until the months preceding the 2020 election, when most voters form their evaluation of incumbents’ performance. Thus, we can only assume that either the observed changes between 2014 and 2017 continued until 2020 (even if at a different pace), or that the changes that occurred between 2017 and 2020 were similarly distributed across all the sampled towns, conditional on the other covariates included in regression models. For all these reasons, we can only provide suggestive evidence, and we encourage further research into the mechanisms that might lead natural threats to influence voting.

## 6 Conclusions

From personal health to material welfare, the COVID-19 pandemic has disrupted human lives at an unprecedented scale. Consistent with research on natural disasters, we find that the threat of COVID-19 has also affected voting behaviour. Focusing on municipal elections that took place in France at the beginning of the pandemic in spring 2020, we find that support for incumbents increased in areas particularly hard hit by the virus. The effect is not sensitive to candidates’ political affiliation, meaning that incumbents from both the left and the right obtained higher vote shares in areas more strongly affected by the pandemic. The results are robust to a placebo test and hold across different methods, including regressions with lagged dependent variables, first-difference regressions, a differences-in-differences approach and propensity score matching.

While previous studies have focused on the aftermath of natural disasters, we provide novel evidence for how voters react to such disasters while they are unfolding, and incumbent politicians cannot be held accountable for the post-disaster management. In this sense, we show that natural threats can boost support for incumbents even in the absence of retrospective evaluations. At the very beginning of the pandemic, it was unclear how to best respond to the circulation of the virus, and local candidates lacked the legal authority to introduce strong containment measures, giving voters little to evaluate. This finding is in line with recent evidence that incumbents’ popularity increased across several countries *before* governments took active measures to reduce the spread of COVID-19 [18, 71].

Setting retrospective evaluations aside, we argue that voters support incumbents as a means to reduce the fear and anxiety generated by the circulation of the virus [13]. We provide indirect evidence for this emotional channel, by showing that votes for incumbents increased, especially in areas where the circulation of the virus was high and people felt particularly anxious about the pandemic. A relevant implication of this mechanism is that incumbents benefit from the presence of external threats simply by virtue of representing the status quo, *regardless* of their performance or political leaning. In this sense, our findings can be interpreted as further evidence of a status-quo bias in voting for political candidates (see [72–75]), which is heightened in the presence of fear-inducing threats [24].

We further argue that a prospective-voting channel can also explain our results. Voters, we reason, believe that incumbents can act more swiftly than challengers against the looming threat of the pandemic, simply because they represent continuity with the current administration. Although we cannot test this mechanism directly, we hypothesise that this mechanism should especially benefit mayors with a positive administrative record. Voters might infer that a candidate who performed well in one domain, might perform equally well in handling the pandemic–even if these domains are unrelated. In line with this reasoning, we find that voters are particularly likely to support incumbents where the threat of COVID-19 is high and welfare improved over the last electoral cycle.

Further research should attempt to clarify the mechanisms underlying the effects of natural threats on support for incumbents in the absence of retrospective evaluations. While we argue that both an emotional channel and a prospective-voting channel might explain such effects, we lack individual-level data that can address these mechanisms directly. We also call for further research into the nature of rally-around-the-flag effects. While our findings are in line with evidence of rally effects at the beginning of the pandemic [17–20, 22, 36–38], we argue that the threat of COVID-19 differs from the type of threats that, according to previous studies, lead people to rally around the flag, such as terrorist attacks and military crises. In particular, COVID-19 should not activate the same feelings of anger and revenge that have been theorized to explain rally effects [29], since the virus lacks the intention to attack a population. Further research should test whether facing a natural threat (such as COVID-19) or a human threat (such as a terrorist attack) is associated to different emotional states of fear or anger, which, in turn, can lead to different behavioural outcomes (for a review, see [43]).

Lastly, we should not overstate the “power” of the incumbency status in influencing voting behaviour. Although the effects we detect can change the outcome of elections, they are likely to be short-lived, in line with research on the effects of terrorist attacks [31] and COVID-19 as well [76]. As the pandemic unfolds, a number of considerations probably influence voters’ decisions, including the evaluation of how incumbents and political representatives responded to the spread of the virus [77]. Indeed, evidence indicates that support for governments in Western democracies has decreased during the course of the pandemic [78]. In this sense, voters are not blind to the performance of their political representatives. After the initial emotional reaction to the spread of COVID-19, it is likely that other considerations shape voting decisions.

## Supporting information

S1 File Supporting information including additional analyses. (PDF)
