# Supplementary material for: How COVID-19 affects voting for incumbents: Evidence from local elections in France
Source: PLoS One. 2024 Mar 19;19(3):e0297432. doi: 10.1371/journal.pone.0297432 (PMC10950211; doi:10.1371/journal.pone.0297432)
Supplement: S1 File — (PDF) [file pone.0297432.s001.pdf]

Supplementary Information

Table S1. Predictors of city with incumbent (vs. no incumbent)
Table S2. Summary statistics
Table S3. Political affiliation of incumbents
Table S4. Effects of COVID-19 on voting for incumbents
Table SS. Additional models introducing covariates gradually
Table S6. First-difference regression models
Table S7. Effects of COVID-19 (alternative specification of COVID-19 spread)
Table S8. Interactions with political affiliation of incumbents
Table S9. Propensity score matching
Table S10. Placebo test: effect of COVID-19 on vote for incumbents in 2014
Table S11. Difference-in-differences (DiD)
Table S12. DiD in regression framework
Table S13. Testing the parallel trend assumption
Table S14. Testing mechanisms with interaction models

Fig S1. Testing mechanisms (alternative specification of COVID-19 spread)
Fig S2. DAG justifying the choice of control variables
Fig S3. Testing the parallel trend assumption
Fig S4. Weekly death count in France between 2001 and 2020
Fig S5. Number of recorded death on each year
Fig S6. Random selection of municipalities with a population larger than 1000
Fig S7. Worst fitting municipalities among the 34972 municipalities
Fig S8. Three measures of COVID-19 severity aggregated at the regional level
Fig S9. Posterior predictive check for the prevalence model (Equation (8))
Fig S10. Correlation between the COVID-19 outbreak severity measure and various measures from the departmental-level hospital dataset
Fig S11. Correlation between the COVID-19 outbreak severity measure and the level of anxiety reported during the first week after the election. Every measure is at the regional level
Fig S12. Correlation between the COVID-19 outbreak severity measure and the log distance between each municipality and the nearest COVID-19 hotspot

A Additional results

Table S1. Predictors of city with incumbent (vs. no incumbent)

	City with incumbent (vs city without incumbent)	
Covid-19 spread (deciles)	-0.008	(0.008)
Population density (logged)	0.132***	(0.030)
Male to female ratio (logged)	0.340	(0.281)
Share of immigrants (logged)	0.052	(0.037)
Share of blue collars (logged)	-0.106	(0.105)
Share of unemployed (logged)	-0.015	(0.107)
Share of aged 65+ (logged)	-0.046	(0.115)
Median household income	-0.003*	(0.001)
Share of vocational degree (logged)	0.026	(0.209)
Share of bachelor degree (logged)	0.089	(0.113)
Baseline mortality (logged)	0.077*	(0.032)
Turnout 2014	1.204***	(0.316)
Number of candidates (2020)	-0.111***	(0.022)
County fixed effects	Yes	
N	8913	
Pseudo-R^2	.020	

Note: Logistic regression of city with incumbent vs city without incumbent.
Coefficients are log-odds with standard errors in parentheses
* p<.05, ** p<.01, *** p<.001

Table S2. Summary statistics

	Count	Mean	SD	Min	Max
Incumbent vote share 2020	4952	72.71	24.93	7.78	100
Incumbent vote share 2014	4952	68.04	22.41	20.66	100
Covid-19 spread	4952	0.01	0.03	0.001	0.99
Covid-19 spread (logged, normalized)	4952	0.27	0.12	0	1
Population density (logged)	4952	5.32	1.22	1.57	9.887
Male to female ratio (logged)	4952	-0.05	0.09	-0.40	0.94
Share of immigrants (logged)	4952	-3.24	0.94	-6.31	-0.69
Share of blue collars (logged)	4952	-2.28	0.40	-4.95	-1.24
Share of unemployed (logged)	4952	-2.21	0.37	-3.92	-0.92
Share of people aged 65+ (logged)	4952	-1.61	0.30	-3.25	-0.66
Median household income (hundreds of euros)	4952	218.83	33.43	133.2	449.9
Share of vocational degree (logged)	4952	3.31	0.21	1.61	3.74
Share of bachelor degree (logged)	4952	2.09	0.36	0.59	2.93
Baseline mortality (logged)	4952	-7.47	0.86	-9.49	-2.23
Turnout 2014	4952	0.67	0.09	0.32	0.98
Number of candidates (first round 2020)	4952	2.05	1.32	1.00	14.00

Note: Summary statistics at the municipality level. Only municipalities with more than 1000 inhabitants and an incumbent candidate in 2020.

Table S3. Political affiliation of incumbents

Political affiliation in 2014	Name of the list	2014 abbreviation	Frequency	Percent
Left-wing	Front de gauche	FG	16	0.32
	Parti de gauche	PG	2	0.04
	Parti communiste français	COM	38	0.77
	Parti socialiste	SOC	267	5.39
	Union de la gauche	UG	208	4.20
	Divers gauche	DVG	1,016	20.52
	Europe-Ecologie-Les Verts	VEC	6	0.12
Centre / Unclear	Modem	MDM	16	0.32
	Union du centre	UC	16	0.32
	Union des démocrates et indépendants	UDI	144	2.91
	Divers	DIV	887	17.91
Right-wing	Les Républicains	UMP	243	4.91
	Union de la droite	UD	205	4.14
	Divers droite	DVD	1,878	37.92
	Rassemblement national	FN	7	0.14
	Extrême droite	EXD	3	0.06
Total			4,952	100

Table S4. Effects of COVID-19 on voting for incumbents

	Vote share for incumbents in 2020 (first round)			
	(1)	(2)	(3)	(4)
Covid-19 spread (deciles)	0.272***			
	(0.082)			
Covid-19 spread (r.c.=1st quart.)				
- 2nd quartile		0.372		
		(0.664)		
- 3rd quartile		1.549*		
		(0.663)		
- 4th quartile		1.722**		
		(0.665)		
Covid-19 spread (logged, normalized)			3.913*	
			(1.960)	
Covid-19 spread (r.c.=1st quint.)				
- 2nd quintile				-0.312
				(0.741)
- 3rd quintile				0.660
				(0.744)
- 4th quintile				1.490*
				(0.737)
- 5th quintile				1.808*
				(0.743)
Population density (logged)	2.443***	2.438***	2.462***	2.454***
	(0.331)	(0.331)	(0.331)	(0.331)
Male to female ratio (logged)	3.387	3.418	3.244	3.447
	(2.998)	(3.000)	(3.001)	(3.000)
Share of immigrants (logged)	0.457	0.456	0.458	0.444
	(0.401)	(0.401)	(0.401)	(0.401)
Share of blue collars (logged)	1.514	1.515	1.502	1.501
	(1.133)	(1.134)	(1.134)	(1.133)
Share of unemployed (logged)	-1.573	-1.580	-1.518	-1.577
	(1.161)	(1.161)	(1.161)	(1.161)
Share of aged 65+ (logged)	-6.867***	-6.879***	-6.953***	-6.880***
	(1.242)	(1.243)	(1.243)	(1.243)
Median income (hundreds euros)	-0.097***	-0.097***	-0.096***	-0.097***
	(0.015)	(0.015)	(0.015)	(0.015)
Share of vocational degree (logged)	-8.628***	-8.646***	-8.562***	-8.604***
	(2.225)	(2.225)	(2.226)	(2.225)
Share of bachelor degree (logged)	3.080*	3.077*	3.064*	3.061*
	(1.229)	(1.230)	(1.230)	(1.230)
Baseline mortality (logged)	1.728***	1.735***	1.766***	1.729***
	(0.344)	(0.344)	(0.344)	(0.344)
Turnout 2014	1.505	1.498	1.705	1.669
	(4.510)	(4.515)	(4.514)	(4.514)
Incumbent vote share 2014	0.186***	0.186***	0.186***	0.187***
	(0.016)	(0.016)	(0.016)	(0.016)
Number of candidates (2020, 1st round)	-14.441***	-14.440***	-14.459***	-14.436***
	(0.252)	(0.252)	(0.252)	(0.252)
County fixed-effects			Yes	
Constant	120.914***	121.602***	121.096***	121.313***
	(10.312)	(10.310)	(10.329)	(10.311)
N	4,952	4,952	4,952	49,52
R^2	0.589	0.588	0.588	0.589

Note: OLS regression coefficients with standard errors in parentheses. Covid-19 spread: excess mortality weighted by age and sex-specific infection fatality ratios. All variables measured at the municipality level. * p<.05, ** p<.01, *** p<.001

Table S5. Additional models introducing covariates gradually

	Vote share for incumbents in 2020 (first round)				
	(1)	(2)	(3)	(4)	(5)
Covid-19 spread (deciles)	0.463***	0.480***	0.279***	0.190***	0.276***
	(0.121)	(0.116)	(0.083)	(0.051)	(0.081)
Baseline mortality	Yes	Yes	Yes	Yes	Yes
Municipality-level covariates	No	Yes	Yes	Yes	Yes
Turnout 2014	No	No	Yes	Yes	Yes
Number of candidates (2020 1st round)	No	No	Yes	No	Yes
Number of candidates categorical	No	No	No	Yes	No
Incumbent vote share 2014	No	No	No	Yes	Yes
County fixed-effects	Yes	Yes	Yes	Yes	No
N	4952	4952	4952	4952	4952
R^2	0.093	0.171	0.577	0.840	0.574

Note: OLS regression coefficients with standard errors in parentheses, introducing covariates gradually. Covid-19 spread: excess mortality weighted by age and sex-specific infection fatality ratios. Number of candidates categorical: number of candidates running at first round of elections, recoded in 3 categories: one candidate, two candidates, three or more candidates. * p<.05, ** p<.01, *** p<.001

Table S6. First-difference regression models

	Vote share for incumbents (difference between 2020 and 2014, first rounds)			
	(1)	(2)	(3)	(4)
Covid-19 spread (deciles)	0.349**	0.345**	0.244*	0.245*
	(0.123)	(0.123)	(0.102)	(0.102)
Baseline mortality	Yes	Yes	Yes	Yes
Municipality-level covariates	No	Yes	Yes	Yes
Turnout 2014	No	No	Yes	Yes
Number of candidates (2020 1st round)	No	No	Yes	Yes
County fixed-effects	Yes	Yes	Yes	No
N	4952	4952	4952	4952
R^2	0.026	0.028	0.338	0.294

Note: OLS regression coefficients with standard errors in parentheses. First-difference regressions of incumbent vote share. Dependent variable: incumbent vote share in 2020 (first round) minus incumbent vote share in 2014 (first round). Covid-19 spread: excess mortality weighted by age and sex-specific infection fatality ratios. * p<.05, ** p<.01, *** p<.001

Table S7. Effects of COVID-19 on voting for incumbents (alternative specification of COVID-19 spread)

	Vote share for incumbents in 2020 (first round)			
	(1)	(2)	(3)	(4)
Covid-19 spread (deciles)	0.233**			
	(0.082)			
Covid-19 spread (r.c.=1st quart.)				
- 2nd quartile		0.046		
		(0.665)		
- 3rd quartile		1.015		
		(0.667)		
- 4th quartile		1.609*		
		(0.664)		
Covid-19 spread (logged, normalized)			3.426†	
			(2.098)	
Covid-19 spread (r.c.=1st quint.)				
- 2nd quintile				1.145
				(0.740)
- 3rd quintile				1.029
				(0.744)
- 4th quintile				1.743*
				(0.741)
- 5th quintile				2.165**
				(0.743)
Municipality-level covariates			Yes	
Baseline mortality			Yes	
Turnout 2014			Yes	
Incumbent vote share 2014			Yes	
Number of candidates 2020			Yes	
County fixed-effects			Yes	
N	4952	4952	4952	4952
R^2	0.588	0.588	0.588	0.588

Note: OLS regression coefficients with standard errors in parentheses. Covid-19 spread: excess mortality (4 weeks after election) weighted by age and sex-specific infection fatality ratios. The models include the same covariates included in Table S4. †, p≤ .1, $*p<.05, **p<.01, ***p<.001$

Table S8. Interactions with political affiliation of incumbents

	Vote share for incumbents in 2020 (first round)			
	(1)	(2)	(3)	(4)
Covid-19 spread (deciles)	0.355*	0.193		
	(0.143)	(0.180)		
Covid-19 spread (r.c.=1st quart.)				
- 2nd quartile			0.517	0.061
			(1.175)	(1.372)
- 3rd quartile			1.499	0.082
			(1.184)	(1.439)
- 4th quartile			2.460*	0.614
			(1.170)	(1.449)
Incumbent (left-wing)	r.c.	-0.375	r.c.	-0.393
		(1.429)		(1.329)
Incumbent (centre/unclear)	0.375	r.c.	0.393	r.c.
	(1.429)		(1.329)	
Incumbent (right-wing)	0.952	0.577	0.393	0.000
	(1.167)	(1.337)	(1.086)	(1.245)
Left-wing incumbent X Covid-19	r.c.	0.162		
		(0.229)		
Centrist incumbent X Covid-19	-0.162	r.c.		
	(0.229)			
Right-wing incumbent X Covid-19	-0.109	0.053		
	(0.185)	(0.214)		
Left-wing incumbent X Covid-19 2nd q.			r.c.	0.456
				0.456
Centrist incumbent X Covid-19 2nd q.			-0.456	r.c.
			(1.802)	
Right-wing incumbent X Covid-19 2nd q.			-0.110	0.346
			(1.525)	(1.685)
Left-wing incumbent X Covid-19 3rd q.			r.c.	1.418
				(1.861)
Centrist incumbent X Covid-19 3rd q.			-1.418	r.c.
			(1.861)	
Right-wing incumbent X Covid-19 3rd q.			0.663	2.081
			(1.517)	(1.724)
Left-wing incumbent X Covid-19 4th q.			r.c.	1.846
				(1.860)
Centrist incumbent X Covid-19 4th q.			-1.846	r.c.
			(1.860)	
Right-wing incumbent X Covid-19 4th q.			-0.742	1.104
			(1.505)	(1.733)
Municipality-level covariates	Yes			
Baseline mortality	Yes			
Turnout 2014	Yes			
Incumbent vote share 2014	Yes			
Number of candidates 2020	Yes			
County fixed-effects	Yes			
N	4952	4952	4952	4952
R^2	0.589	0.589	0.589	0.589

Note: OLS regression coefficients with standard errors in parentheses. The models include the same covariates included in Table S4. * p<.05, ** p<.01, *** p<.001

Table S9. Propensity score matching

	Vote share for incumbents in 2020 (first round)	
	(1)	(2)
Covid-19 spread (deciles)	0.273**	
	(0.089)	
Covid-19 spread (r.c.=1st quart.)		
- 2nd quartile		0.924
		(0.731)
- 3rd quartile		1.838*
		(0.721)
- 4th quartile		1.812*
		(0.724)
Municipality-level covariates	Yes	
Baseline mortality	Yes	
Turnout 2014	Yes	
Incumbent vote share 2014	Yes	
Number of candidates 2020	Yes	
County fixed-effects	Yes	
N	4084	4084
R^2	0.595	0.595

Note: OLS regression coefficients with standard errors in parentheses. Sample of matched municipalities. The models include the same covariates included in Table S4. * p<.05, ** p<.01, *** p<.001

Table S10. Placebo tests: Effect of COVID-19 on voting for incumbents in 2014

	Vote share for incumbents in 2014 (first round)	
	(1)	(2)
Covid-19 spread (deciles)	0.052	
	(0.244)	
Covid-19 spread (r.c.=1st quart.)		
- 2nd quartile		1.394
		(2.115)
-3rd quartile		2.428
		(2.172)
-4th quartile		0.228
		(1.997)
Incumbent vote share 2008	0.289**	0.286**
	(0.108)	(0.108)
Municipality-level covariates	Yes	
Baseline mortality	Yes	
County fixed-effects	Yes	
N	533	533
R^2	0.215	0.218

Note: OLS regression coefficients with standard errors in parentheses. Dependent variable: vote share for incumbents in 2014 (first round). The models include the same covariates included in Table S4 (apart from turnout in 2014 and number of candidates in 2020). * p<.05, ** p<.01, *** p<.001

Table S11. Difference-in-differences (DiD)

	Election		Diff
Treatment status	2014	2020	
Control	68.46	72.23	3.77
Treated	67.62	73.19	5.57
Diff	-0.84	0.96	1.80

Note: Raw incumbent vote shares in treated vs. untreated municipalities in 2014 vs. 2020. Treatment defined as being in the third or fourth quartile of municipalities affected by COVID-19.

Table S12. DiD in regression framework

	Vote share for incumbents in 2020 (first round)	
	(1)	(2)
Treated towns	-0.843	0.059
	(0.635)	(0.603)
2020 (vs. 2014) election	3.764***	13.655***
	(0.493)	(0.787)
Treated×2020 election	1.810**	1.804**
	(0.681)	(0.697)
Share of immigrants (logged)		-3.191***
		(0.301)
Share of blue collars (logged)		11.640***
		(0.866)
Share of unemployed (logged)		-14.378***
		(1.047)
Median household income		0.001***
		(0.000)
Constant	68.463***	32.617***
	(0.458)	(2.439)
N	9904	9901
R^2	0.010	0.117

Note: Regression coefficients with bootstrapped standard errors in parentheses. Treatment defined as being in the third or fourth quartile of municipalities affected by Covid-19. * p<.05, ** p<.01, *** p<.001

Table S13. Testing the parallel trend assumption

	Vote share for incumbents	
	(1)	(2)
High Covid-19 spread	-0.125	-0.568
	(1.958)	(1.821)
Year 2008 (vs. 2014)	-2.053	-2.053
	(1.487)	(1.496)
Year 2020 (vs. 2014)	1.539	1.539
	(1.869)	(1.880)
High spread X year 2008	-0.605	-0.605
	(2.211)	(2.224)
High spread X year 2020	2.299	2.299
	(2.481)	(2.496)
Municipality-level covariates	No	Yes
N	612	612
R2	0.020	0.079

Note: Regression coefficients with standard errors clustered at the municipality level in parentheses. Sub-sample of municipalities with same mayor in 2008, 2014 and 2020 (N=204). High Covid-19 spread defined as being in the third or fourth quartile of municipalities affected by Covid-19. * p<.05

Table S14. Testing mechanisms

	Vote share for incumbents in 2020 (first round)		
	(1)	(2)	(3)
Covid-19 spread (deciles)	-0.039	-0.001	0.429*
	(0.244)	(0.149)	(0.168)
Perceived anxiety (0-1)	-1.253		
	(3.500)		
Covid-19 X anxiety	0.702		
	(0.608)		
Change in median income		-0.135	
		(0.111)	
Covid-19 X income change		0.036*	
		(0.017)	
Change in unemployment rate			0.669*
			(0.274)
Covid-19 X unemployment change			-0.031
			(0.029)
County fixed-effects	No	Yes	Yes
Municipality-level covariates	Yes	Yes	Yes
Baseline mortality	Yes	Yes	Yes
Turnout 2014	Yes	Yes	Yes
Incumbent vote share 2014	Yes	Yes	Yes
Number of candidates 2020	Yes	Yes	Yes
Living in rural or urban area	Yes	No	No
N	4820	4952	4952
R^2	0.631	0.589	0.589

Note: OLS regression coefficients with standard errors in parentheses. Column 1: standard errors clustered at the level of municipality-size within county, controlling also for municipality size (below 2000 inhabitants, between 2000 and 20,000, and above 20,000). Change in median household income: hundreds of euros. The models include the same covariates included in Table S4. * p<.05, ** p<.01, *** p<.001

B Additional illustrations

Fig S1. Testing mechanisms (alternative specification of COVID-19 spread)

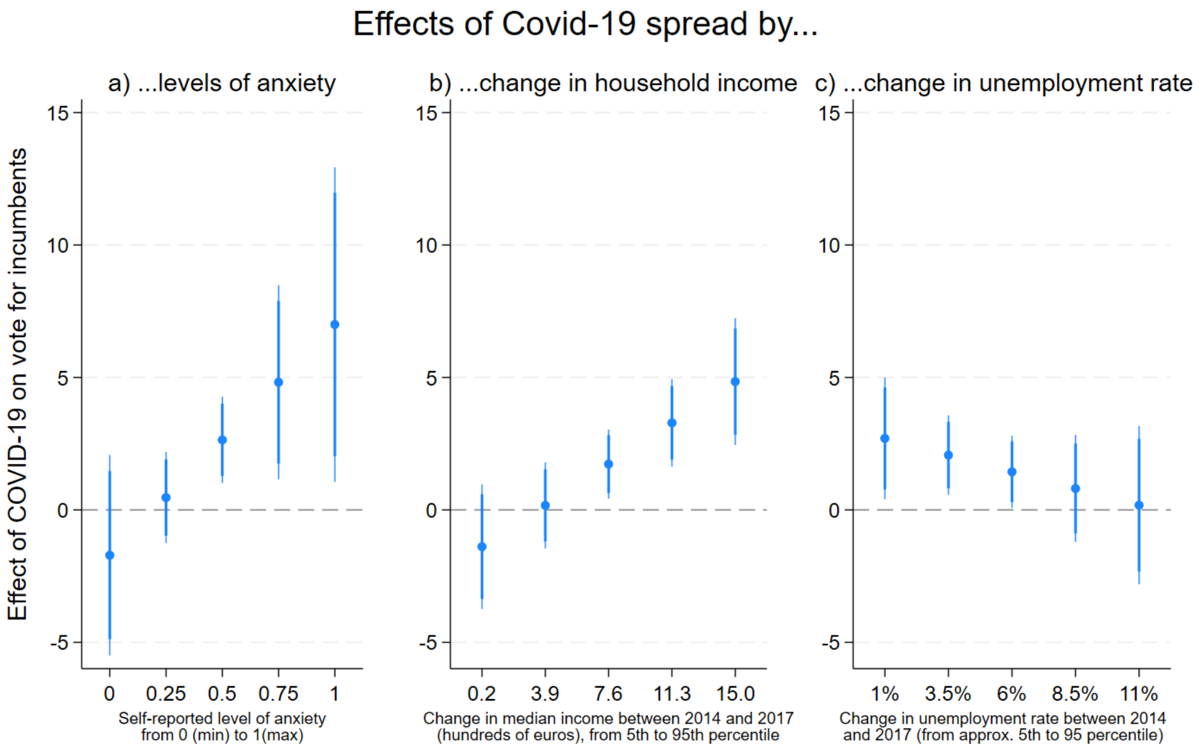

Note: Effect of 4th quartile of COVID-19 spread vs. 1st quartile (equal to value of 0) by interacted variables specified on the X-axes. Coefficients plots from OLS regressions controlling for vote for incumbents in 2014 and additional municipality-level covariates. Thin/thick lines are 95%/90% confidence intervals. Plot a: Effects by average levels of anxiety in 305 geographical units (municipalities of similar size within counties).

Fig S2. DAG justifying the choice of control variables

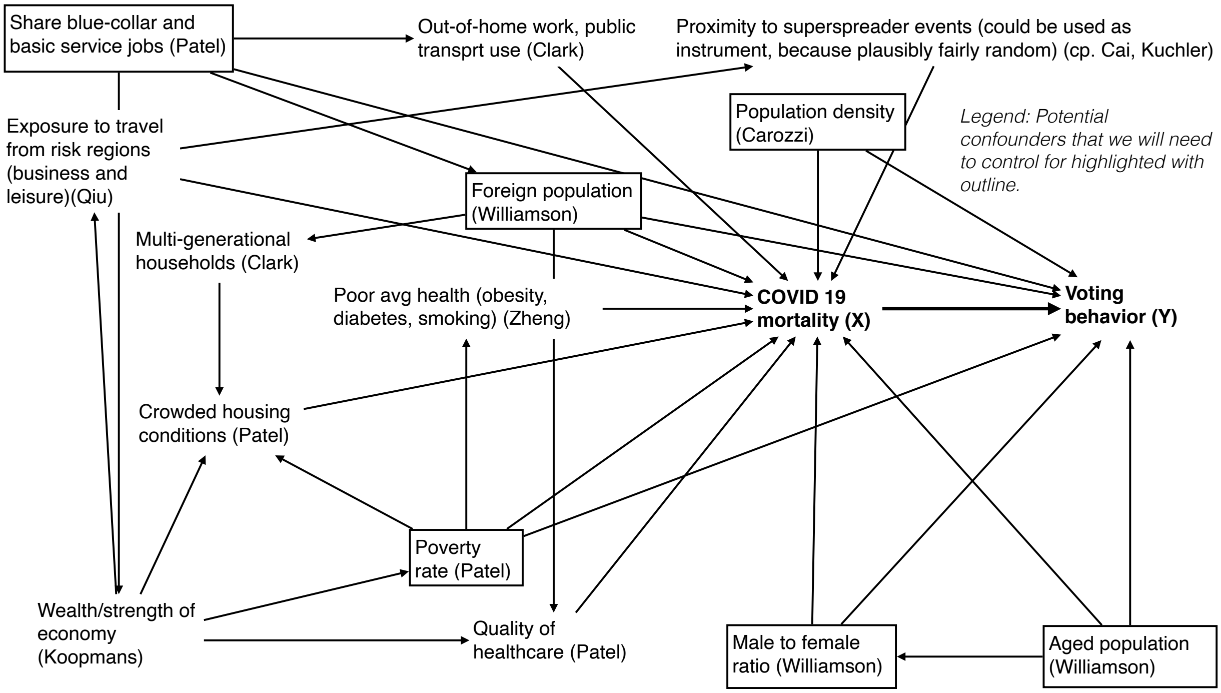

Note: Directed acyclic graph (DAG) showing the interrelationship between factors predicting mortality from COVID-19 and factors directly affecting incumbent support. Potential confounders highlighted with a black outline. We control for these factors in our regression model. Names in the diagram refer to the following publications: [SI1, SI2, SI3, SI4, SI5, SI6, SI7, SI8]

Fig S3. Testing the parallel trend assumption

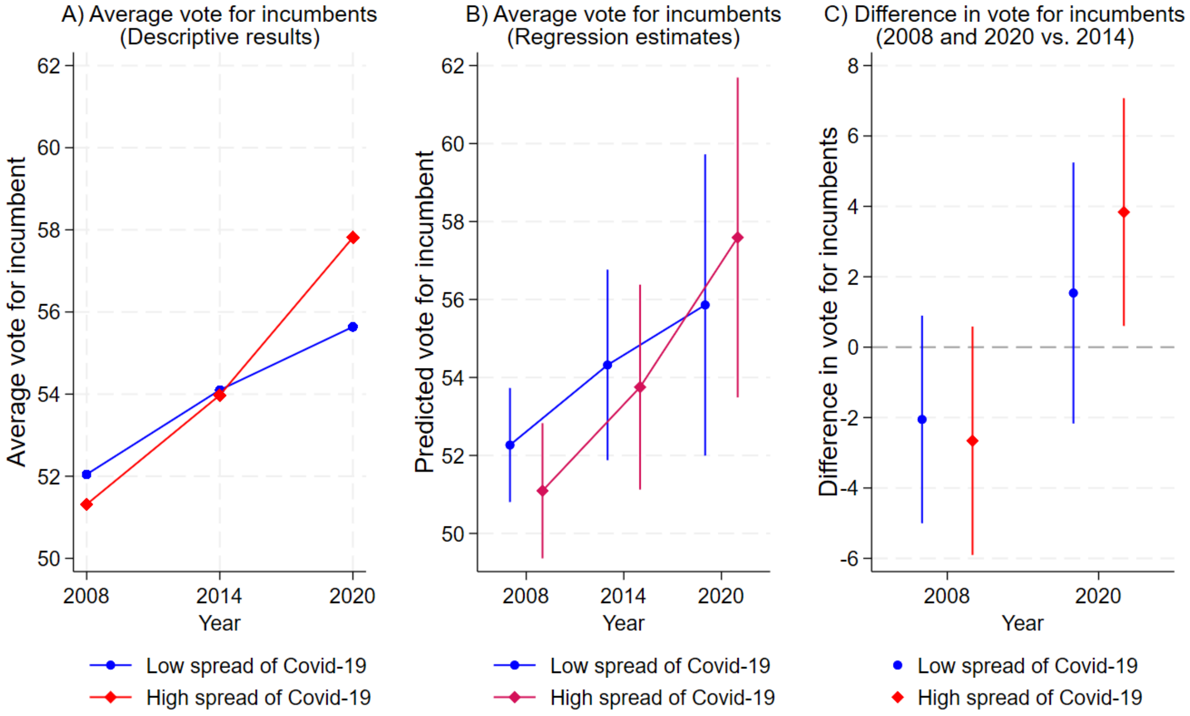

Note: Sub-sample of municipalities with same mayor in 2008, 2014 and 2020 (N=204). Low/high Covid-19 spread defined as being in the first or second quartile (low) or in the third or fourth quartile (high) of municipalities affected by Covid-19. Plots B and C based on regression model 2 in Table S13.

C Estimating the spread of COVID-19

C.1 Aggregate mortality figures

Fig S4. Weekly death count in France between 2001 and 2020, as of April 14th 2020

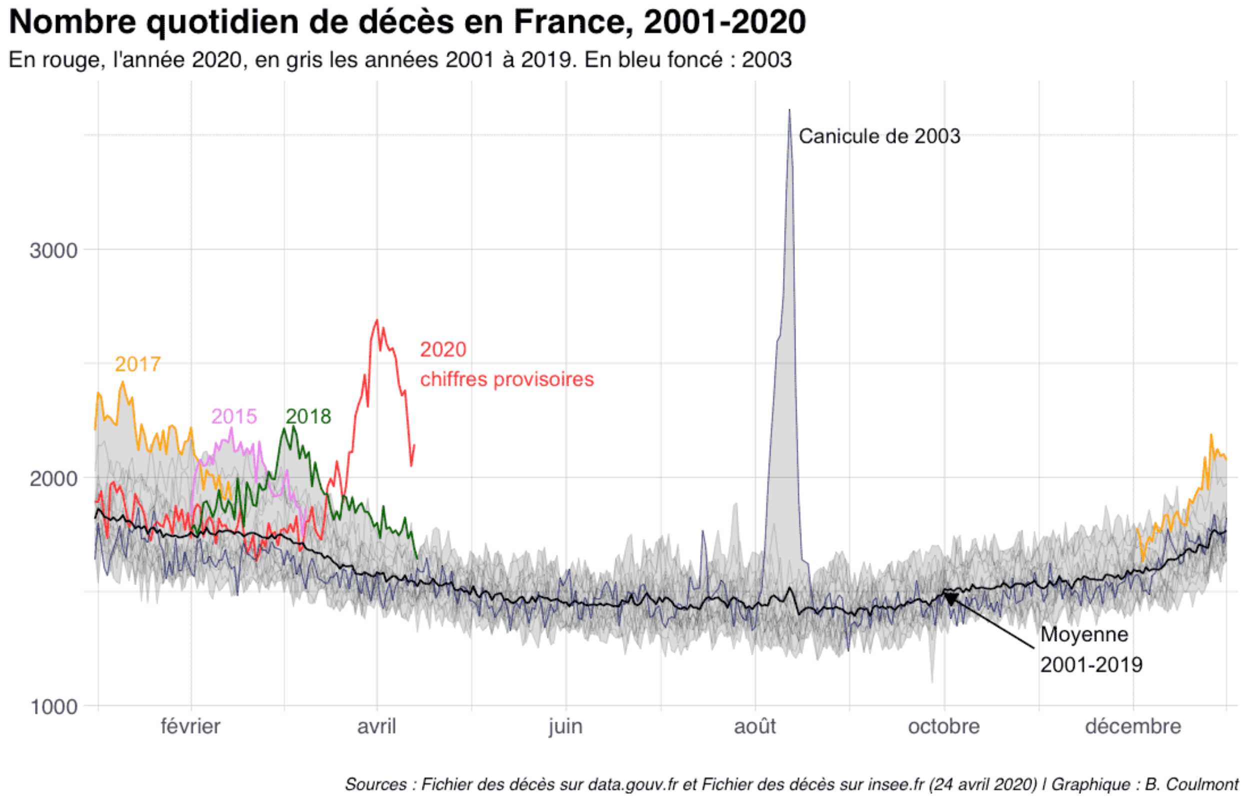

Note: Large peaks are clearly visible and correspond to extreme mortality events: the 2003 heat wave, yearly flu outbreaks and the 2020 pandemic. Mortality records from data.gouv.fr and from INSEE.fr, image by B. Coulmont.

Fig S5. Number of recorded death on each year, aggregated at country level and during the period of interest.

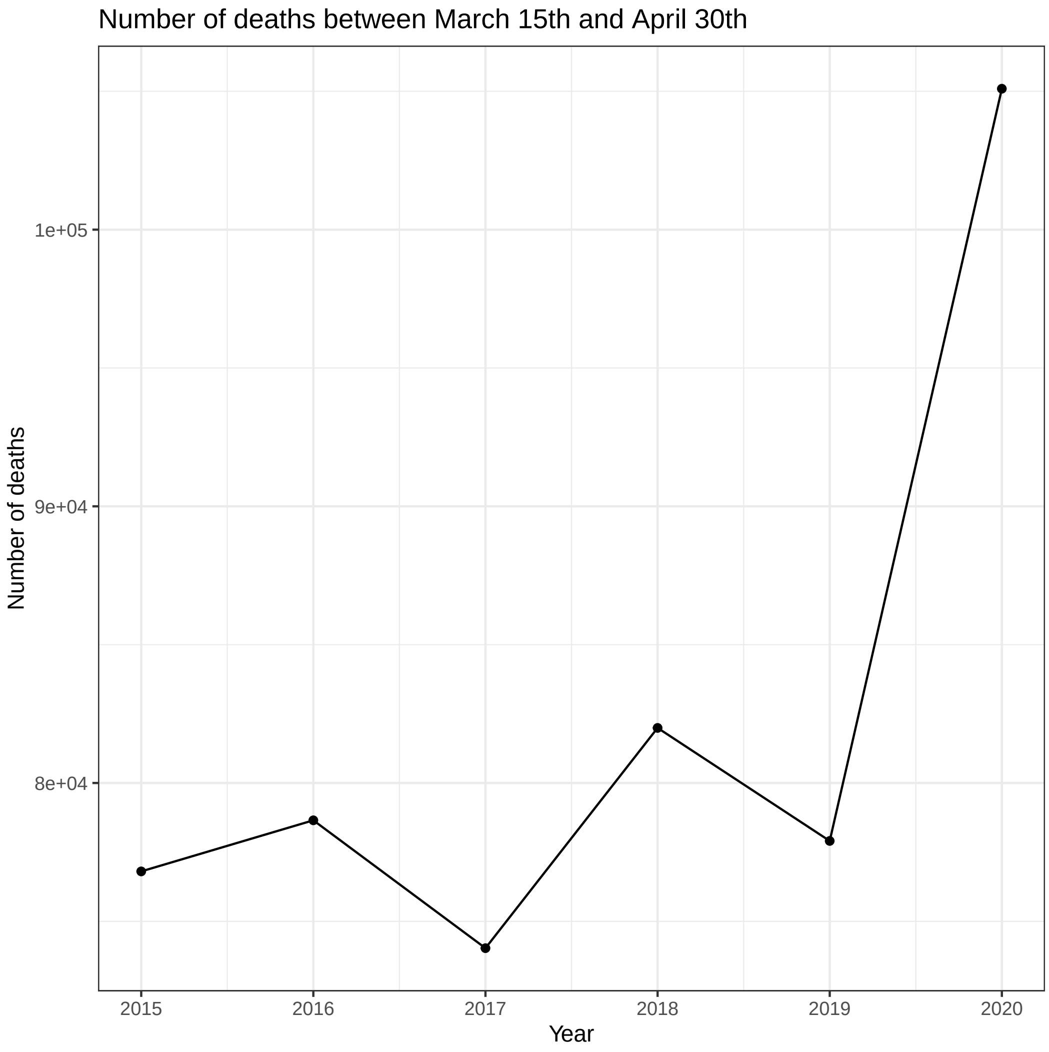

C.2 Population extrapolation

Population data at the municipality level is only available with a 3-year lag[1]. To obtain population data for 2018, 2019 and 2020, we extrapolated the population from the previous years by estimating the municipality-specific evolution trend over the years 2010-2017. We added up the population of the municipalities that merged in 2020 but were distinct at some point in the past.

We consider three basic extrapolation models:

- constant population
- linear increase with time
- linear increase with time on the log scale

To predict the population in 2019 and 2020 for each municipality, we aim to use the best model according to a parsimony criterion and a goodness of fit criterion. More precisely, this can be formalised in the following four models. Let N_{mt} be the population of municipality m at year t:

$$N_{mt} = N_m + \epsilon \quad (2)$$
$$\log N_{mt} = \log N_m + \epsilon \quad (3)$$
$$N_{mt} = \alpha + \beta t + \epsilon \quad (4)$$
$$\log N_{mt} = \alpha + \beta t + \epsilon \quad (5)$$
$$\epsilon \sim \text{Normal}(0, \sigma) \quad (6)$$

For our parsimony criterion, we deem reasonable to rank the models by increasing complexity using the following order: 1) constant population (Equation (2)), 2) constant population on the log scale (Equation (3)), 3) linear trend (Equation (4)) 4) linear trend on the log scale (Equation (5)).

For each model and each municipality, our goodness of fit criterion is the Akaike Information Criterion (AIC, [SI9]). A difference in AIC of 3 is sometimes considered as reasonable evidence in favor of one of the two models ([SI10]). When the difference between the best model according to the AIC criterion and a simpler model according to the complexity criterion is smaller than 3, we prefer the simpler model. Therefore, our mixed criteria model selection procedure consists of choosing the simplest model within a radius of 3 of the best model according to the AIC. We also considered the Bayesian Information Criterion [SI9] instead of AIC and obtained similar results.

For the 34972 municipalities considered, the constant model (Equation (2)) was chosen 8 times (0.02%), the log-constant model (Equation (3)) 5559 times (16%) and the log-linear model 29405

[1] Source: https://www.insee.fr/fr/statistiques/1893204, data downloaded on 24th September 2020

Fig S6. Random selection of municipalities with a population larger than 1000

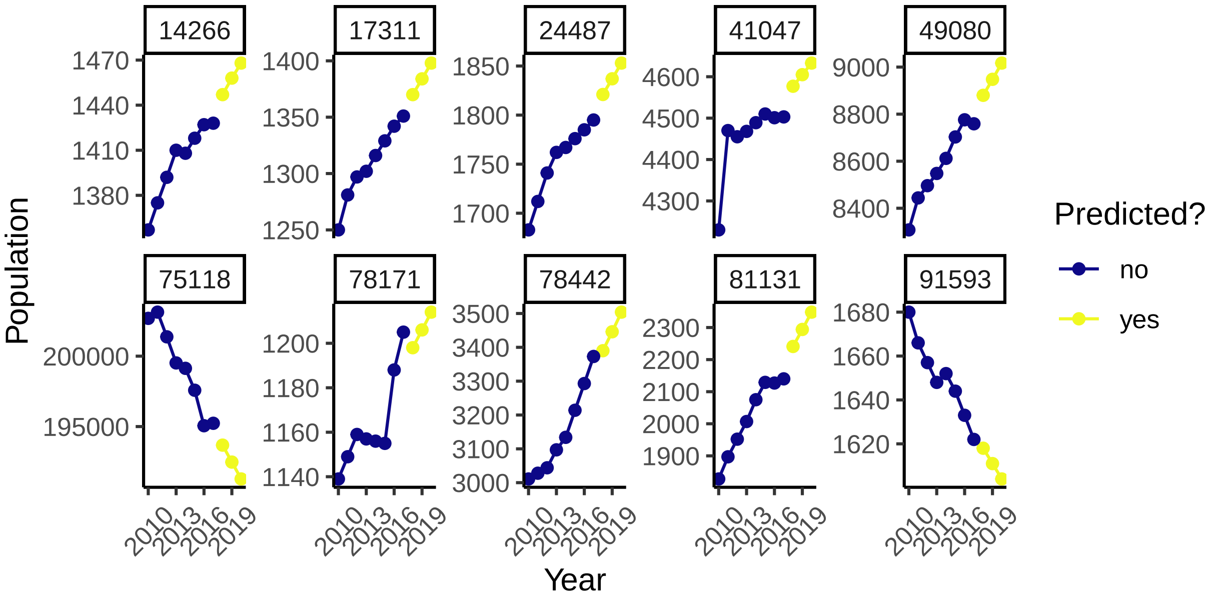

Note: The dark points indicate observed data, the light points indicate extrapolated data. The panel label denotes the INSEE code for the municipality considered.

times (84%). Fig 6 shows a random sample of the municipalities with the data used for fitting the extrapolation model and the extrapolation. In all cases, the extrapolation seems reasonable. Fig 7 shows the cities where the goodness of fit of the selected model, measured by R^2, are the worst among the 34972 municipalities. Even in this worst-case scenario, we see that the extrapolation is not predicting unrealistic values, thus confirming the reliability of our approach.

To obtain population estimates in age and sex categories for each municipality, we use the detailed 2017 census data[2] to estimate the proportion of the population in each age and sex category for each municipality. We then assume these proportions to remain constant between 2015 and 2020 and redistribute the total population of the municipalities at each year in each age and sex category according to these proportions, rounding to the nearest integer.

[2]https://www.insee.fr/fr/statistiques/1893204

Fig S7. Worst fitting municipalities among the 34972 municipalities

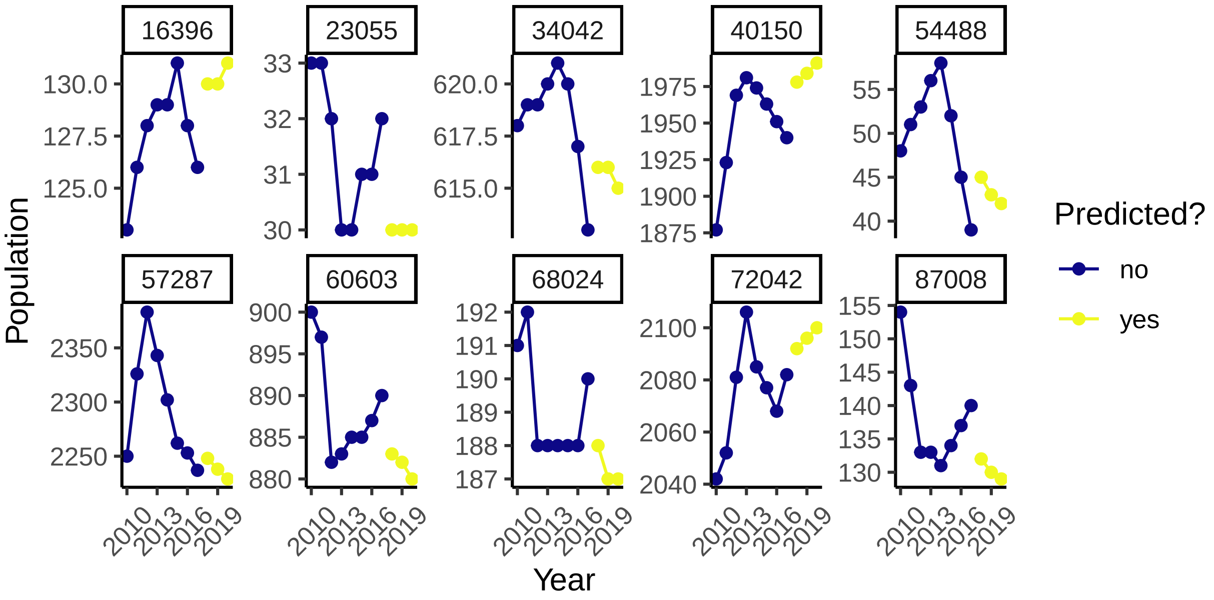

Note: The dark points indicate observed data, the light points indicate extrapolated data. The panel label denotes the INSEE code for the municipality considered.

C.3 Estimating the COVID-19 threat from excess mortality data

Excess mortality models

We model excess mortality in 2020 compared to the previous years through a Poisson model with municipality-specific intensities. We first introduce a basic model for excess mortality which does not take into account the age of the deceased. The purpose of the basic model is to show the added value of the main model, which accounts for the dependence on age and sex of the probability to die once infected (Infection Fatality Ratio, IFR).

For the basic model, let Y_{mt} denote the number of deaths between March 15th and six weeks later in municipality m at year t, and let N_{mt} denote the population of that municipality on that year t. We make the common assumption (see for instance [SI12, 14]) that mortality follows a Poisson distribution with a baseline-municipality specific hazard h_m for $2015 \leq t \leq 2019$ plus a municipality-specific excess hazard h_m^+ for $t = 2020$:

$$
\begin{aligned}
Y_{mt} &\sim \text{Poisson}(N_{mt}h_m) \text{ if } t \neq 2020 \\
Y_{mt} &\sim \text{Poisson}\left(N_{mt}(h_m + h_m^+)\right) \text{ if } t = 2020
\end{aligned}
\tag{7}
$$

In this model, h_m^+ characterises the severity of the COVID-19 outbreak in the municipality. A natural consequence of this model is that the excess probability to die from COVID-19 is the same for every person in any given municipality, which we know to be an oversimplification of reality. We therefore introduce a model accounting for the differential probability of dying from COVID-19 infection depending on age and sex, which has been estimated using data from various countries in [14].

Starting from the mortality records, we attribute each death record to a 5-year age-class matching the classes defined by [14]. We therefore use the best age resolution available for the age-specific Infection Fatality Rate.

We discard death records for children under 10 and for people over 80 because excess death in these age classes can be linked to a variety of causes and are not very informative about the level of COVID-19 spread (see notably the dispersion of Infection Fatality Rate for young children and people over 80 on Figure 1.b. of [14]).

Let Y_{mtas} denote the number of deaths between March 15th and six weeks later in municipality m at year t in age class a for sex s, and let N_{mtas} denote the population in that age and sex class of that municipality at year t. As in the previous section, we assume that mortality follows a Poisson distribution with a baseline municipality-age-sex specific hazard h_{mas} for $t \in 2015 : 2019$ plus a municipality specific excess hazard h_{mas}^+ for $t = 2020$, where the excess hazard involves the COVID-19 prevalence in the municipality ν_m and the age-specific Infection Fatality Ratio ρ_{as}.

We define an age-class continuous variable x_a indicating the index of the age-class (which corresponds to a binning of the age, thus proportional to age) which we scale and center. We further define a sex-class binary variable x_s where the value 1 corresponds to males. These quantities are combined together in the following model:

$$\begin{aligned} Y_{mtas} &\sim \text{Poisson}(N_{mtas}, h_{mas}) \text{ if } t \neq 2020 \\ Y_{mtas} &\sim \text{Poisson}(N_{mtas}, h_{mas} + h_{mas}^+) \text{ if } t = 2020 \\ \log h_{mas} &= \log h_m + \beta_{age} x_a + \beta_{sex} x_s \\ h_{mas}^+ &= \nu_m \rho_{as} \end{aligned} \tag{8}$$

h_m is the baseline hazard in municipality m for females in the central age-class. β_{age} and β_{age} are the country-wide age and sex effects on the baseline mortality.

Note that in this model the prevalence ν_m is a municipality-level quantity, which is the same for all age and sex classes. We use ν_m to characterize the severity of the COVID-19 outbreak in municipality m.

Prior structure

A striking feature of the excess hazard due to COVID-19 in 2020 is that in the period considered, only a few hotspots of COVID infection were scattered around the country, which implies that most municipalities were not affected yet. As a consequence, it is realistic for most h_m^+ and ν_m values to have a negligible value.

We include this insight in the model by performing inference using the Bayesian framework and specifying a sparsity-inducing Lasso prior [SI13] h_m^+ and ν_m, and resorting to Maximum a Posteriori (MAP) estimation. We use the following prior structure for the first model, *not* accounting for the age and sex-specific IFR:

$$\begin{aligned} h_m &\sim \log\mathcal{N}(\mu, \sigma) \\ h_m^+ &\sim \exp(\lambda) \\ \mu &\sim \mathcal{N}(-5, 5) \\ \sigma &\sim \mathcal{N}^+(1, 20) \\ \lambda &\sim \text{Gamma}(0.001,\ 0.001) \end{aligned} \tag{9}$$

where $\mathcal{N}$ denotes the Normal distribution, $\mathcal{N}^+$ denotes the truncated Normal distribution on the positive real numbers and $\log\mathcal{N}^+$ denotes the lognormal distribution.

We use the following prior structure for the second model which accounts for the age and sex-specific IFR:

$$\begin{aligned} h_m &\sim \log\mathcal{N}(\mu, \sigma) \\ \nu_m &\sim \exp(\lambda) \\ \mu &\sim \mathcal{N}(-5, 5) \\ \sigma &\sim \mathcal{N}^+(1, 20) \\ \lambda &\sim \text{Gamma}(0.001,\ 0.001) \\ \beta_{age}, \beta_{sex} &\sim \mathcal{N}(0, 2) \end{aligned} \tag{10}$$

We use very vague priors on the location and scale parameters for the baseline mortality. For h_m^+ and ν_m we chose a prior on $\mathbb{R}^+$ with positive mass at 0 to allow for shrinkage of the MAP to 0. Rather than using cross-validation to select the optimal penalty parameter, we follow the recommendations of [SI13] and [SI14] and use a Gamma distribution for the Lasso parameter λ, although we place this prior on λ rather than λ^2 since we do not have conjugacy constraints.

This is essentially the same prior structure for both models, with for the second model a weakly

informative prior on the age and sex effect on the baseline mortality. Since the sex covariate is binary and the age covariate is scaled and centred, the priors allows everything from negligible to large positive or negative effects.

Inference

Given the large number of municipalities, the previous models have a large number of parameters, on the order of 60 000. This renders standard Monte Carlo Markov Chain inference very time-consuming. Since the goal of this analysis is to obtain point estimates for the municipality-level excess hazard or the municipality-level prevalence, we resort to Variational Inference, which is known for good performance in obtaining point estimates for a posterior distribution [SI15]. We use a stochastic variational inference algorithm [SI16] through the interface available in Stan [SI17]. Sparsity is obtained by calculating MAP values conditional on the optimal value of the penalty parameter λ, estimated by the median marginal posterior distribution on λ. Optimisation of the posterior to obtain the MAP is performed using the default Limited-memory Broyden–Fletcher–Goldfarb–Shanno (LBFGS) algorithm implementation in Stan.

C.4 Estimated values and validation

Posterior predictive model checking

Fig 8 compares the two estimations of COVID-19 outbreak severity to a naive estimate, the empirical excess hazard, aggregated at the regional level. The empirical excess hazard is obtained by dividing the number of deaths by the population size for each year, then subtracting to the empirical hazard for 2020 the mean hazard for the previous years. This empirical estimate does not take into account population size and is thus sensitive to outliers. The model-based excess hazard (Equation (7)) and the prevalence (Equation (8)) are more robust because they take into account the size of the population in the Poisson intensity and they benefit from the country-wide shrinkage (Equations (9) and (10)) for the baseline mortality estimates and from the lasso penalty.

This increased robustness is expected to play a role at the municipality level, where populations may be small and fluctuations large. At the regional level, with larger aggregated population, the model-based estimate of excess hazard and the empirical estimates should be close, and this is indeed what we see on Fig 8, where we see large excess hazard in the Grand-Est region and the Île-de-France region, as expected.

Since the prevalence measure takes into account the age structure of the population, it can provide different values depending on the age at death of the people on the mortality records. Indeed, we see that our assessment of the severity of the COVID-19 outbreak is fairly different from the basic

excess hazard model ((Equation (7))), with the Île-de-France region now much more severely affected than the other regions. Because of the comparatively older population in the Grand-Est region, a COVID-19 outbreak comparable to other regions caused a large excess mortality. Conversely, the prevalence measure reveals that although excess hazard was not the highest in the Île de France region, the COVID-19 outbreak was particularly severe, given the markedly younger population in counties such as Seine-Saint-Denis.

We perform posterior predictive checks for our main model (Equation (8)), where we predict the number of deaths for each municipality based on our MAP estimates and compare them with the observed number of deaths. Fig 9 shows that predictions based on the MAP estimates generally perform well. The 95% prediction intervals mostly cover the true value, with an average coverage of 99% , which suggests that the data is slightly under-dispersed compared to a Poisson error model.

Validation by comparison with external measures

We compare our measure of the spread of COVID-19 (prevalence) with other measures obtained from publicly available data. These measures are rarely available at the municipality level and may require aggregating prevalence at the county or regional level. First, we use county-level data from hospitals, such as the number of hospitalised people, the number of people in intensive care or the number of deceased per inhabitant.[3] These measures should be relatively noise-free, assuming that the quality of hospital reporting is high, but they are still partial, as they document only the gravest cases of COVID-19 infection. These data contain information closely related to excess death, and we observe a correlation with our prevalence measure (Fig 10).

Second, we retrieve data from the CoviPrev survey[4], which tracked the evolution of mental health and general behaviour – such as the adoption of preventative measures – in the French population since March 23, 2020. These data are recorded at the regional level. Fig 11 shows a strong correlation between the spread of Covid-19 and anxiety at the regional level, lending support to our measure of the level of threat perceived by the voters.

Third, we use data compiled by [55] who estimate the spread of COVID-19 using the distance to the COVID-19 hotspots known to be active at the time of the election. This measure can be compared directly with our measure since it is available at the municipality level. As expected, our measure of prevalence is negatively correlated with the log-distance of the municipalities to the nearest COVID-19 hostpot (Fig 12).

Since our measure of prevalence can be imprecise for municipalities with a small population,

[3] Source: data.gouv.fr. Available at https://www.data.gouv.fr/fr/datasets/donnees-hospitalieres-relatives-a-lepidemie-de-covid-19/

[4] Source:https://www.santepubliquefrance.fr/etudes-et-enquetes/coviprev-une-enquete-pour-suivre-l-evolution-des-comportements-et-de-la-sante-mentale-pendant-l-epidemie-de-COVID-19)

Fig S8. Three measures of COVID-19 severity aggregated at the regional level

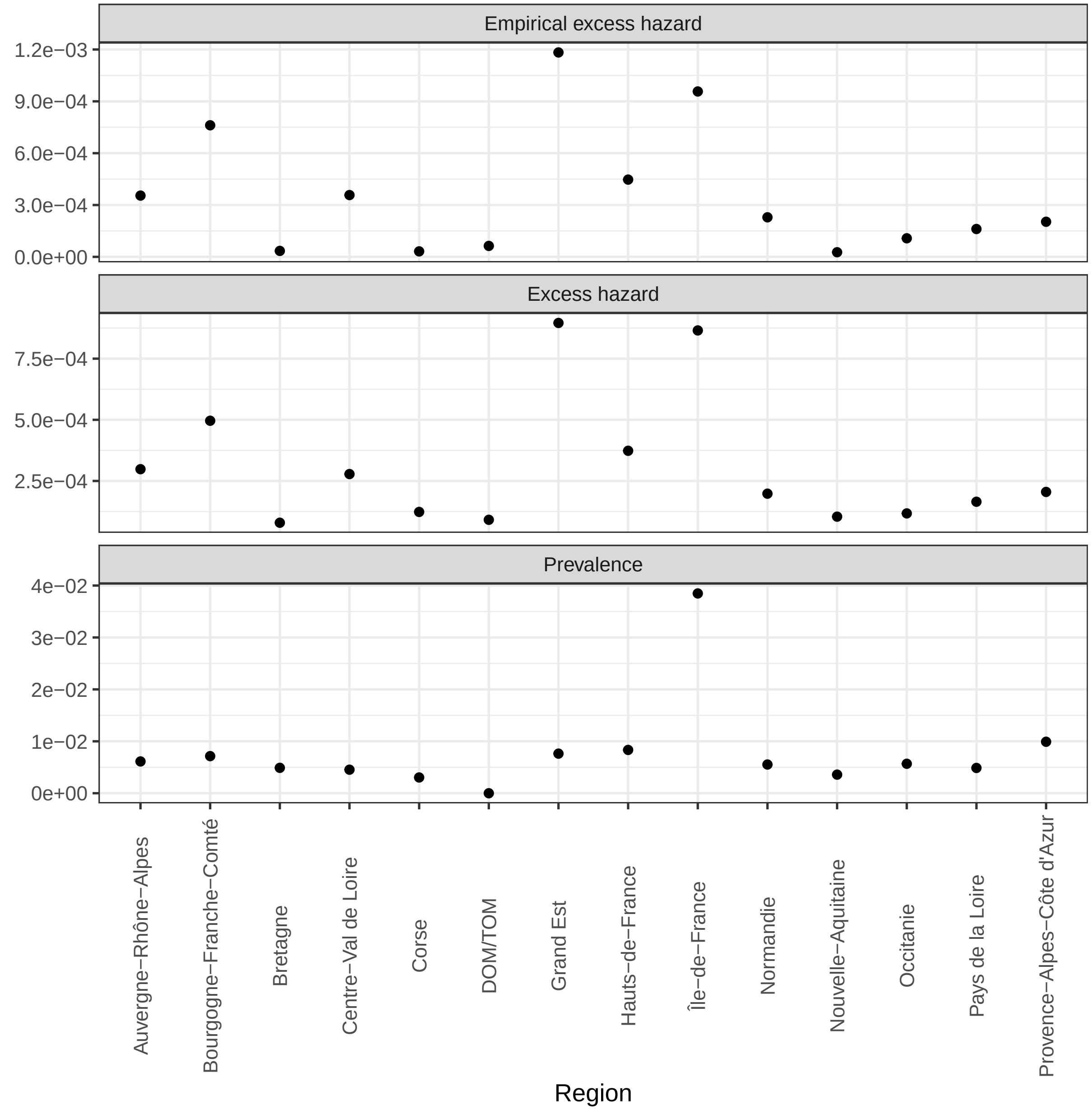

Fig S9. Posterior predictive check for the prevalence model (Equation (8))

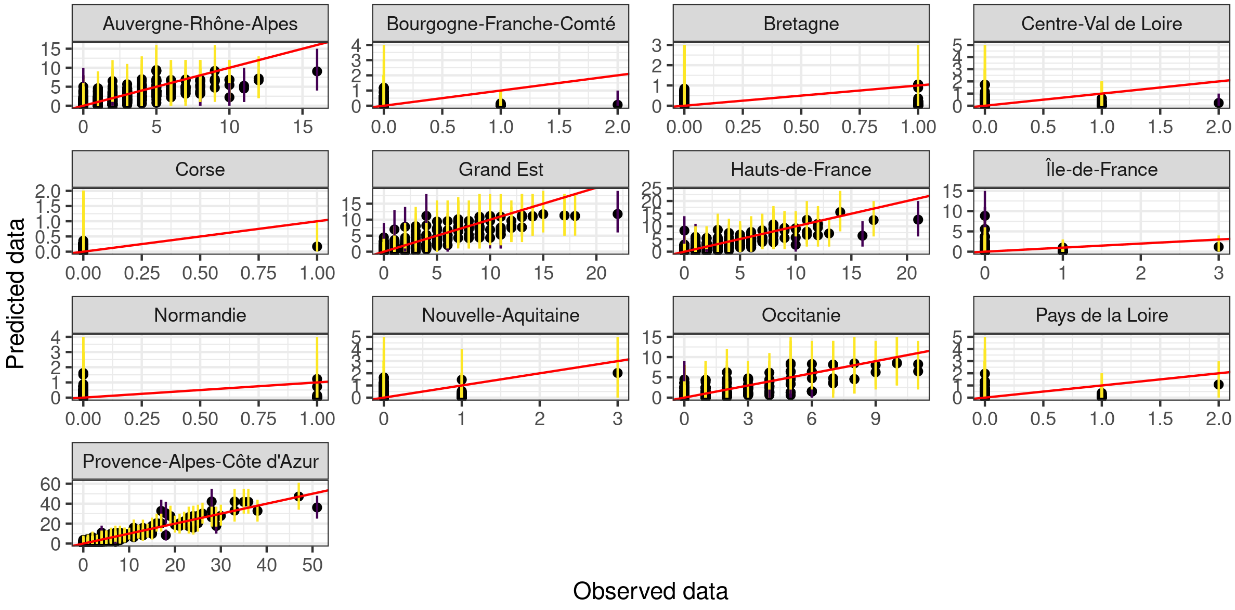

Note: The mean predictive estimate for the number of death in each age and sex category, for each municipality and each year is plotted against the observed number of deaths. The prediction is performed using the MAP estimates for the parameters. The vertical segments denote the 95% prediction interval; they have a light colour if they cover the observed data and a dark colour if they do not. The identity line is denoted in red.

Fig S10. Correlation between the COVID-19 outbreak severity measure and various measures from the departmental level hospital dataset.

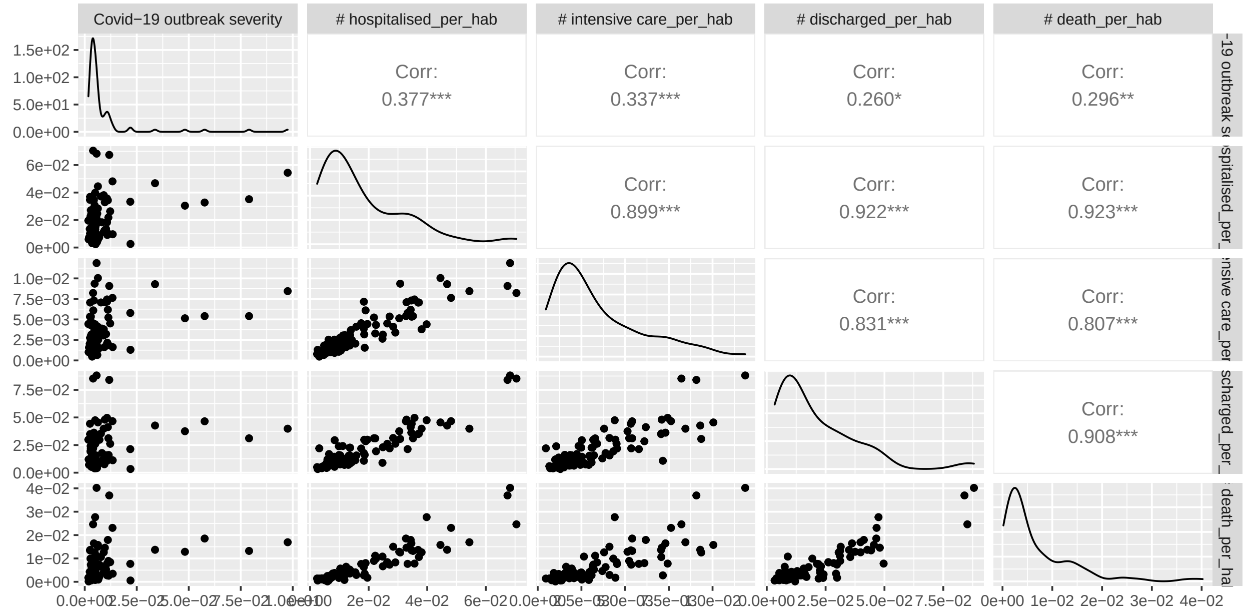

if we remove all municipalities with a population below 10000, we find a marked increase in the magnitude of the correlation (Fig 12, bottom).

Fig S11. Correlation between the COVID-19 outbreak severity measure and the level of anxiety reported during the first week after the election. Every measure is at the regional level.

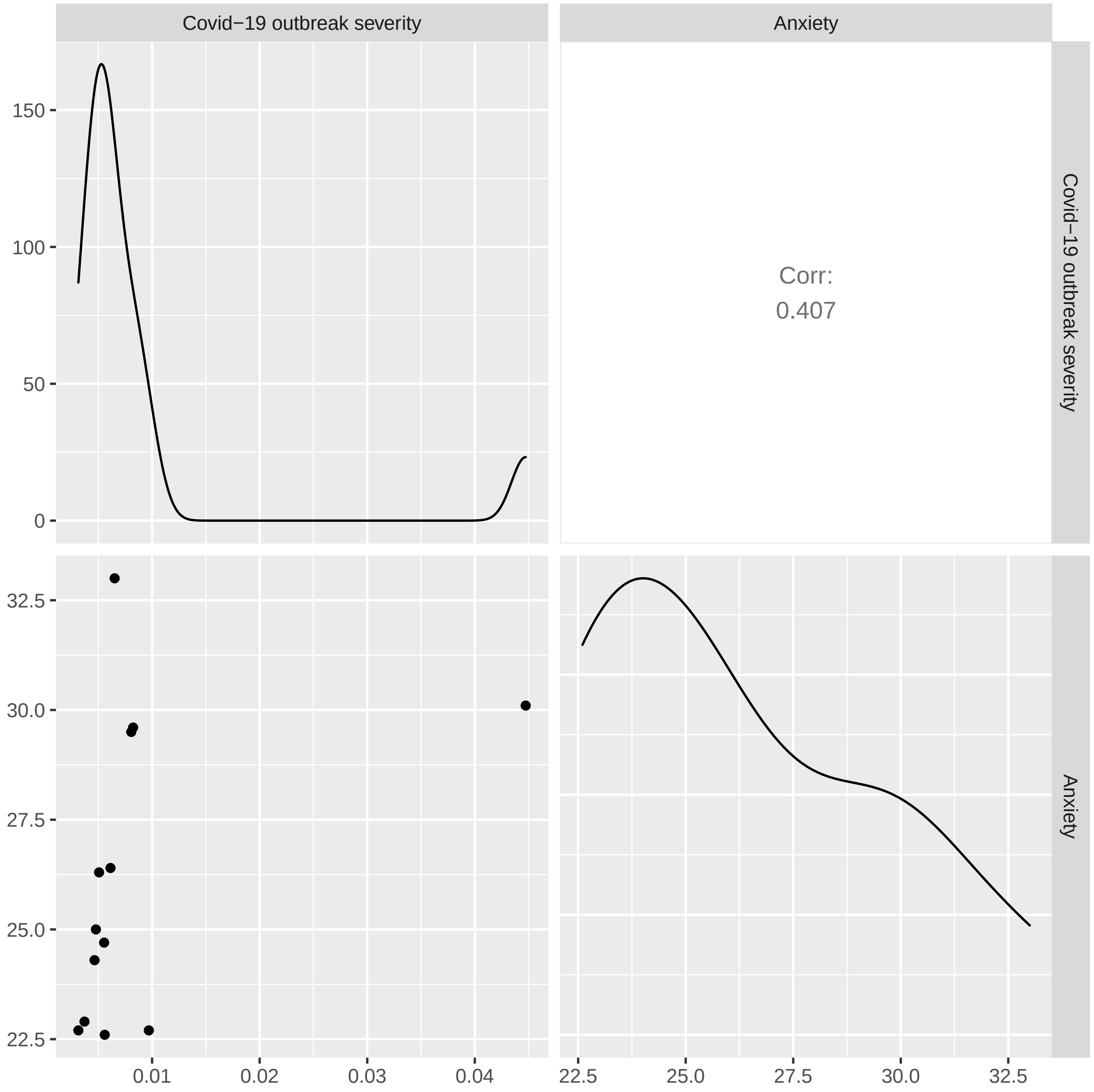

Fig S12. Correlation between the COVID-19 outbreak severity measure and the log-distance between each municipality and the nearest COVID-19 hotspot

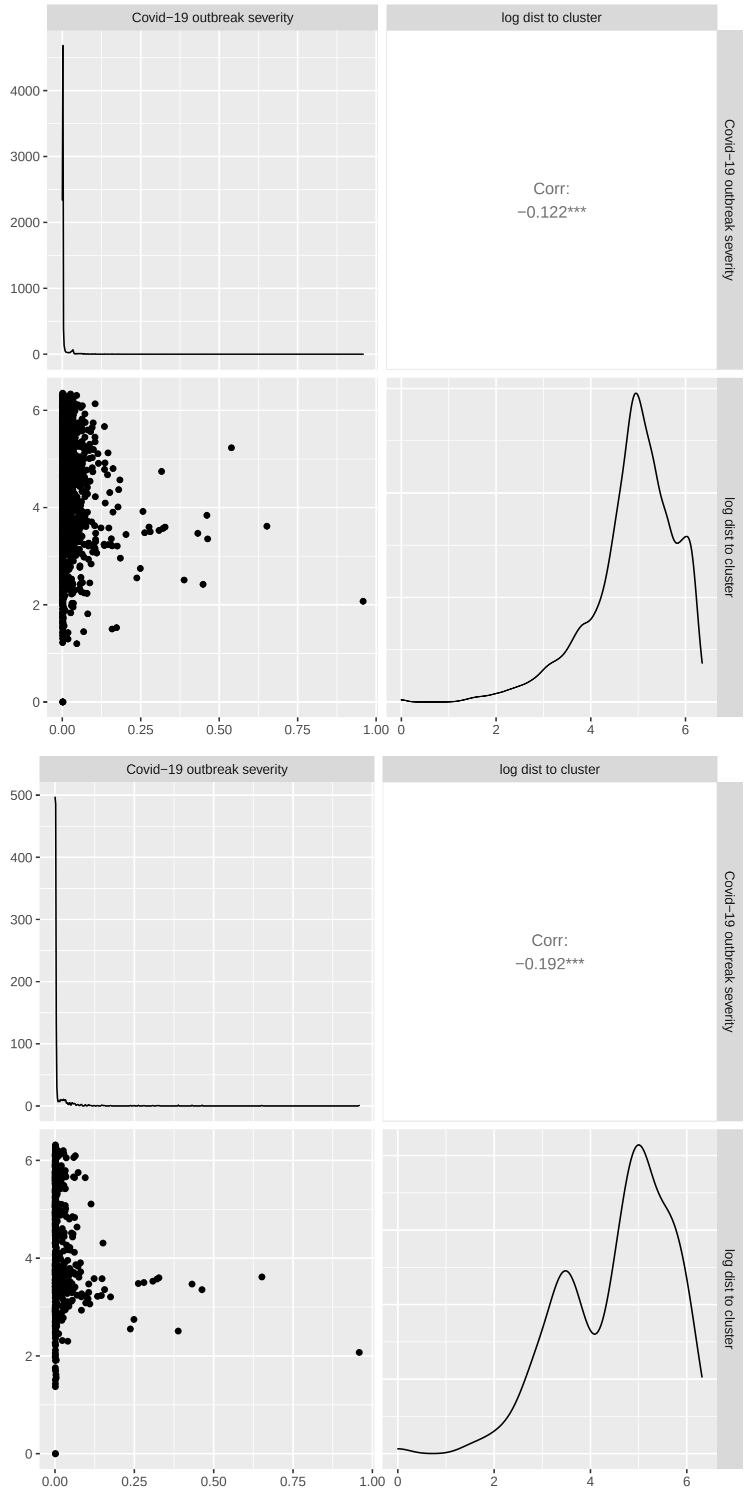

Note: Log-distance as measured in [55]. The bottom figure is the same correlation, computed only on municipalities with a population larger or equal to 10000.

References SI

[SI1] Cai Y, Goehring G. The 2020 Sturgis Motorcycle Rally and COVID-19; 2020.

[SI2] Carozzi F. Urban Density and Covid-19. Rochester, NY: Social Science Research Network; 2020. ID 3643204.

[SI3] Clark E, Fredricks K, Woc-Colburn L, Bottazzi ME, Weatherhead J. Disproportionate Impact of the COVID-19 Pandemic on Immigrant Communities in the United States. PLOS Neglected Tropical Diseases. 2020;14(7):e0008484.

[SI4] Koopmans R. A Virus That Knows No Borders? Exposure to and Restrictions of International Travel and the Global Diffusion of COVID-19. WZB Discussion Paper; 2020.

[SI5] Kuchler T, Russel D, Stroebel J. The Geographic Spread of COVID-19 Correlates with the Structure of Social Networks as Measured by Facebook. National Bureau of Economic Research; 2020. 26990.

[SI6] Patel JA, Nielsen FBH, Badiani AA, Assi S, Unadkat VA, Patel B, et al. Poverty, Inequality and COVID-19: The Forgotten Vulnerable. Public Health. 2020;183:110–111.

[SI7] Qiu Y, Chen X, Shi W. Impacts of Social and Economic Factors on the Transmission of Coronavirus Disease 2019 (COVID-19) in China. Journal of Population Economics. 2020;: 1.

[SI8] Williamson EJ, Walker AJ, Bhaskaran K, Bacon S, Bates C, Morton CE, et al. Factors Associated with COVID-19-Related Death Using OpenSAFELY. Nature. 2020;584(7821, 7821):430–436.

[SI9] Sakamoto Y, Ishiguro M, Kitagawa G. Akaike Information Criterion Statistics. Dordrecht, The Netherlands: D Reidel. 1986;81(10.5555):26853.

[SI10] Burnham KP, Anderson DR, Huyvaert KP. AIC Model Selection and Multimodel Inference in Behavioral Ecology: Some Background, Observations, and Comparisons. Behavioral Ecology and Sociobiology. 2011;65(1):23–35. doi:10.1007/s00265-010-1029-6.

[SI11] O'Driscoll M, Ribeiro Dos Santos G, Wang L, Cummings DAT, Azman AS, Paireau J, et al. Age-Specific Mortality and Immunity Patterns of SARS-CoV-2. Nature. 2021;590(7844):140–145. doi:10.1038/s41586-020-2918-0.

[SI12] Blangiardo M, Cameletti M, Pirani M, Corsetti G, Battaglini M, Baio G. Estimating Weekly Excess Mortality at Sub-National Level in Italy during the COVID-19 Pandemic. PLOS ONE. 2020;15(10):e0240286. doi:10.1371/journal.pone.0240286.

[SI13] Park T, Casella G. The Bayesian Lasso. Journal of the American Statistical Association. 2008;103(482):681–686. doi:10.1198/016214508000000337.

[SI14] Casella G, Ghosh M, Gill J, Kyung M. Penalized Regression, Standard Errors, and Bayesian Lassos. Bayesian Analysis. 2010;5(2):369–411. doi:10.1214/10-BA607.

[SI15] Blei DM, Kucukelbir A, McAuliffe JD. Variational Inference: A Review for Statisticians. Journal of the American Statistical Association. 2017;112(518):859–877.

[SI16] Kucukelbir A, Ranganath R, Gelman A, Blei DM. Automatic Variational Inference in Stan. arXiv preprint arXiv:150603431. 2015;.

[SI17] Stan Development Team. Stan Modeling Language Users Guide and Reference Manual; 2021.
